# MSCs Conditioned Media and Umbilical Cord Blood Plasma Metabolomics and Composition

**DOI:** 10.1371/journal.pone.0113769

**Published:** 2014-11-25

**Authors:** Tiago Pereira, Galya Ivanova, Ana Rita Caseiro, Paula Barbosa, Paulo Jorge Bártolo, José Domingos Santos, Ana Lúcia Luís, Ana Colette Maurício

**Affiliations:** 1 Departamento de Clínicas Veterinárias, Instituto de Ciências Biomédicas de Abel Salazar (ICBAS), Universidade do Porto (UP), Porto, Portugal; 2 Centro de Estudos de Ciência Animal (CECA), Instituto de Ciências e Tecnologias Agrárias e Agro-Alimentares (ICETA), Porto, Portugal; 3 REQUIMTE, Departamento de Química e Bioquímica, Faculdade de Ciências, Universidade do Porto, Porto, Portugal; 4 Biosckin, Molecular and Cell Therapies S.A. TecMaia, Maia, Portugal; 5 CDRsp- Centro para o Desenvolvimento Rápido e Sustentado de Produto, Instituto Politécnico de Leiria, Marinha Grande, Portugal; 6 CEMUC, Departamento de Engenharia Metalúrgica e Materiais, Faculdade de Engenharia, Universidade do Porto, Porto, Portugal; French Blood Institute, France

## Abstract

Human mesenchymal stem cells (hMSCs) from umbilical cord (UC) blood (UCB) and matrix are tested clinically for a variety of pathologies but *in vitro* expansion using culture media containing fetal bovine serum (FBS) is essential to achieve appropriate cell numbers for clinical use. Human UCB plasma (hUCBP) can be used as a supplement for hMSCs culture, since UCB is rich in soluble growth factors and due to worldwide increased number of cryopreserved UCB units in public and private banks, without the disadvantages listed for FBS. On the other hand, the culture media enriched in growth factors produced by these hMSCs in expansion (Conditioned medium - CM) can be an alternative to hMSCs application. The CM of the hMSCs from the UC might be a better therapeutic option compared to cell transplantation, as it can benefit from the local tissue response to the secreted molecules without the difficulties and complications associated to the engraftment of the allo- or xeno-transplanted cells. These facts drove us to know the detailed composition of the hUCBP and CM, by ^1^H-NMR and Multiplexing LASER Bead Technology. hUCBP is an adequate alternative for the FBS and the CM and hUCBP are important sources of growth factors, which can be used in MSCs-based therapies. Some of the major proliferative, chemotactic and immunomodulatory soluble factors (TGF-β, G-CSF, GM-CSF, MCP-1, IL-6, IL-8) were detected in high concentrations in CM and even higher in hUCBP. The results from ^1^H-NMR spectroscopic analysis of CM endorsed a better understanding of hMSCs metabolism during *in vitro* culture, and the relative composition of several metabolites present in CM and hUCBP was obtained. The data reinforces the potential use of hUCBP and CM in tissue regeneration and focus the possible use of hUCBP as a substitute for the FBS used in hMSCs *in vitro* culture.

## Introduction

### hMSCs secretome evaluation and impact in biomedical applications

As demonstrated in some studies, grafted cells usually do not remain in the wound for a long period. In addition, they do not translocate to other regions throughout the body, suggesting that their role is largely limited to signaling which initiates the recruitment and direction of endogenous cells and by growth factors production [1], [2]. Cell signaling is a complex process of communication between different cells and forms the basis of all cellular activities. Proliferation, differentiation, migration, and apoptosis are all processes instructed by different signals [3]. Nowadays it is becoming particularly important to understand the comprehensive characterization of hMSCs secretome, as the factors secreted by these cells seem to be primarily responsible for their therapeutic action [4]. The hypothesis that the location where cells grow and expand in culture (so called conditioned media) could be an appropriate therapeutic product rich in growth factors comparable to hMSCs local application, seemed to be a rational approach to our study [5]. MSCs were found to produce and secrete multiple paracrine factors with therapeutic relevance for their anti-oxidants, anti-apoptotic, anti-fibrotic, angiogenic, immunomodulatory and chemoattractive activities [2], [6], [7]. As already described before by [6], culture supernatants of MSCs (derived umbilical cord Wharton's jelly like the cells used in our study) present several cytokines and other secreted factors such as interleukin type 2 (IL-2), IL-6. IL-8, IL-12, IL-15, monocyte chemotactic protein-1 (MCP-1), macrophage inflammatory protein- 1 beta (MIP-1β), chemokin (C-C motif) ligand 5 (RANTES) and platelet-derived growth factor – AA (PDGF-AA). It should be kept in mind that MSCs might suffer a change in their secretory profile when exposed to an immunoreactive environment [4]. This fact was not considered in the present study since the secretory profile of these cells was only evaluated *in vitro*. There might be some relevant differences in terms of the presence or levels of some cytokines, when comparing the effect of CM to direct transplantation of MSCs in the site of injury. Carvalho et al. [4] have demonstrated that the secretome of the infused umbilical cord blood (UCB) MSCs can modulate the action of central nervous system (CNS) cells, which could be important in tissues with such a low regenerative potential. Fraga et al. [8] revealed that the secretome of the perivascular umbilical cord MSCs (HUCPVCs) improved cell viability, proliferation and neuronal cell densities when *in vitro* tested with neurons isolated from different brain regions which can be useful in patients with spinal cord injury (SCI) and brain ischemia.

### The importance of umbilical cord blood plasma (hUCBP) in mesenchymal stem cells (hMSCs) cryopreservation, in vitro culture and expansion

MSCs as defined by the International Society for Cellular Therapy (ISCT) in 2006, are cells characterized by: a) their capacity to adhere to plastic; b) expression of specific surface markers, namely, CD73, CD90, and CD105, and no expression of CD14, CD19, CD34, CD45 and HLA-DR. Additionally, according to the ISCT, MSCs are able to undergo tri-lineage differentiation into adipocytes, chondrocytes and osteoblasts [9]. Human MSCs (hMSCs) are nowadays, one of the most promising types of stem cells for cell-based therapies. As a matter of fact these cells based on their differentiation capacity, hematopoietic support as well as their immunomodulatory and pro-regenerative properties, have been tested in a large number of clinical trials for treatment of several pathologies like brain paralysis, SCI, cardiovascular diseases and myocardial infarction, type I diabetes, multiple sclerosis, Crohn's disease, bone fractures, graft-*versus*-host disease (GVHD) in bone marrow transplantation, osteoarthritis and rheumatoid arthritis [10]. Currently 369 clinical trials are found only by searching for “mesenchymal stem cells”. If we introduce as key words “mesenchymal stem cells” and “cord tissue”, we found only 3 clinical trials recruiting patients in http:/clinicaltrials.gov. In the same internet site from the U.S. National Institutes of Health, there are 24 clinical trials listed searching for “mesenchymal stem cells” and “umbilical cord blood” (accessed June 2014).

At the moment it is possible to isolate the hMSCs from various sources like bone marrow, adipose tissue, skeletal muscle, umbilical cord blood (UCB), umbilical cord tissue or matrix (UCT, Wharton's jelly), peripheral blood, dental pulp, and amniotic fluid [11]. Also the hMSCs *in vitro* expansion is essential to achieve appropriate cell numbers for clinical use and the *in vitro* culture must be scale-up for clinical application purposes. Some of the complications in preparing hMSCs for cell-based therapies are due to the inconsistent cell culture protocols and the obtained number of viable cells, so the hMSCs *in vitro* culture must be scale-up for clinical application purposes. Since the studies performed by Friedenstein and collaborators in 1970 [12], fetal bovine serum (FBS) and other animal sera have been used for culture media supplementation. Because the animal sera have several disadvantages including economic, ethical and scientific ones, it is important to define alternatives for hMSCs *in vitro* culture. These include chemically defined media, human serum, UCB serum or plasma (hUCBS or hUCBP), human platelet lysate (HPL) and platelet-rich plasma (PRP) [11]. As a matter of fact, the use of FBS is always associated with batch-to-batch variability, unexpected cell growth characteristics, cytotoxicity of uncharacterized factors in the serum, and risk of possible contamination with virus, prions, bacteria, nanobacteria, mycoplasma, yeast, fungi, and endotoxins [13]–[16]. Also, the use of FBS implies a huge number of fetal calves that must be euthanized for blood collection. This does not follow the 3R's policy (reducing, replacing and refining) that should be applied in research [17]. There is also a concern for FBS clinical use, due to the immunogenicity of the FBS cultured cells, inducing anaphylactic or arthus-like immune reactions which were already reported in some patients infused with *in vitro* cultured lymphocytes [15], [16]. Anyway, FBS-cultured hMSCs have been approved by the US Food and Drug Administration (FDA) for use in several human clinical trials mentioned previously. hMSCs are not capable of surviving in the absence of FBS, not only because of the important growth factors present, but also the fact that the FBS works as a buffering agent and protects the cells from cytotoxic agents. These characteristics must also be present in any alternative to animal serum used to isolate and expand these cells. The substitute medium supplement must have a low cost, be safe in terms of contaminants and readily/easily available.

The most attractive alternative to FBS is the hUCBP (allogenic or autologous) due to worldwide increase in the number of cryopreserved cord blood units in Public and Private Cord Blood Banks. The UCB has been used as an important source of hematopoietic stem cells for transplantation in the treatment of hematologic disorders and malignancies [18], [19]. Using hUCBP as a supplement for hMSCs culture and cryopreservation is an important alternative because UCB is rich in soluble growth factors that support the growth, proliferation, and differentiation of resident stem cell populations present in fetal blood [20]. The hUCBP has albumin, transferrin, and fibronectin in high abundance, both very important for cell culture and proliferation. Other growth factors and proteins are present, like epidermal growth factor (EGF), fibroblast growth factor (FGF), nerve growth factor (NGF), vascular endothelial cell growth factor (VEGF), insulin-like growth factors (IGFs), transforming growth factors (TGFs), interferons and interleukins [11]. These factors are involved in cell proliferation, differentiation and migration. Also, some of these factors, like the NGF, regulate the apoptosis. Others like some interleukins are responsible for the maintenance of stemness [21], [22]. The quantification and characterization of these factors present in the hUCBP is important in order to be used in the cryopreservation of UCT and hMSCs isolated from the UCT. UCB, and more recently UCT, have been stored cryopreserved in Private and Public Cord Blood and Tissue banks worldwide in order to obtain hematopoietic stem cells and hMSCs [23]. In order to use the hMSCs isolated cells from fresh and cryopreserved UCT in cell-based therapies, the use of autologous hUCBP as culture medium supplement in the cryopreservation process of isolated hMSCs or of the UCT, and *in vitro* proliferation of hMSCs, motivated us to know in a more detailed way, the composition of the hUCBP as well as the CM where the hMSCs are expanded, by means of NMR spectroscopy and by Multiplexing LASER Bead Technology (Eve Technologies, Canada). ^1^H-NMR allows for *ex vivo* and *in vivo* identification of metabolites [24]. Multiplexing LASER Bead Technology (Eve Technologies, Calgary, Alberta, Canada) using the bead analyzer Bio-Plex 200 (Bio-Rad) allows the quantification of numerous analyte categories such as cytokines, chemokines and growth factors which can test simultaneously multiple targets in a single assay. The hypothesis that the CM where these cells grow and expand in culture (CM) could be an appropriate therapeutic product rich in growth factors comparable to hMSCs local application, seemed to be a rational approach to our study. Thus, two different CM were also analyzed by these two methods.

### NMR technique

Nuclear magnetic resonance (NMR) represents a valuable application in terms of monitoring metabolic changes in disease or in response to treatment and the *ex vivo* analysis of biological samples through high-resolution NMR appears also to be one of the most powerful analytical techniques available for metabolic profiling [25]–[28]. A wide range of analytical platforms is increasingly being utilized either alone or in conjunction with NMR spectroscopy to detect and characterize a wider range of metabolites. Such platforms include capillary electrophoresis and high-performance or ultra-performance liquid chromatography, which are coupled with mass spectrometry (MS) [29], [30]. NMR-based metabolomics provides a robust and stable analytical platform with excellent analytical and biological reproducibility, and it was assumed that could be suitable for the proteomic characterization of hMSC secretome and for hUCBP analysis. One of the advantages of ^1^H-NMR over other methods, is that it can generate a large quantity of information concerning the metabolic composition of samples (its spectrum represents the global profile of the analyzed sample), making possible the simultaneous identification and quantification of structurally diverse metabolites, without the need for individual isolation or no special sample preparation. [30], [31]. The main drawback of this technique is the relatively low sensitivity. Additionally, ^1^H NMR spectra of biological systems (tissues, cells, biofluids) are usually extremely complex because of the large number of components in the samples, resulting in spectra with complex line shapes and significant overlap of the resonance signals.

The assignment of the resonance signals in NMR spectra and identification of the metabolites in biological samples may involve a number of spectral techniques, information from databases of known metabolite spectra and NMR assignment software. Though very complex, ^1^H-NMR spectra of some biological samples allow direct assignment of resonance signals of some metabolites based on their chemical shifts, multiplicity and intensity and spectral analysis of appropriate two dimensional (2D) NMR spectra. 2D NMR spectroscopy can be useful to increase the signal dispersion in spectra with significant overlap of the resonances and reveal the connectivity between signals (^1^H/1H Correlation spectroscopy (COSY); ^1^H/^1^H Total Correlation spectroscopy (TOCSY), ^1^H/^13^C or ^1^H/^15^N Hetero-correlation spectroscopy (HSQC, HMBC)) and/or to define the multiplicity and coupling pattern of resonances (2D J- resolved spectroscopy), thereby helping to identify metabolites in biological samples. When integrated with the appropriate software tools for (semi-)automated peak identification, NMR spectral libraries of metabolites promise a significant advance in the interpretability of NMR data [32].

^1^H-NMR based metabonomics and metabolomics have already been used for human embryonic stem cells (hESCs) conditioning media (CM) characterization, proving to be an accurate and valuable tool for monitoring, controlling and optimizing hESC culture media preparation. On the other hand, proteomic/secretome studies performed on hMSCs from the Wharton's jelly umbilical cord are still sparse [4]. Also with this purpose, a number of studies have attempted to identify protein factors secreted by mouse and human fibroblasts produced during the conditioning process [33]. Characterization of cell culture media by ^1^H-NMR has also facilitated the detailed scrutiny of metabolites involved in a variety of biochemical pathways for potential tissue engineering applications [28].

In our study NMR spectroscopy is used as a tool to identify and quantify the metabolites (quantitative and qualitative NMR analysis) in the different biological samples. By NMR spectroscopy (^1^H and ^13^C NMR, application of 1D and 2D (^1^H/^1^H homonuclear and ^1^H, ^13^C heteronuclear correlation techniques), the NMR-observable metabolites have been defined and from there the metabolic profile of the samples. The quantitative distribution of these metabolites in the different samples has been evaluated from ^1^H-NMR spectra.

### Aims of the experimental work

Recent efforts to characterize biomolecule production by stem cells have utilized genomic and proteomic approaches to analyze cell-conditioned media [2]. The present study aimed to present a qualitative and quantitative assessment of the metabolic profile of Wharton's jelly hMSCs conditioned media (CM) in different culture conditions. A commercial media (Promocell) of unknown composition and another common basal medium (DMEM) currently used during hMSCs *in vitro* expansion were used for the process of conditioning. Upon reaching a cell confluence of 80%, the culture media was collected at two different time points (24 and 48 h) in order to assess differences in the metabolic activity of these cells during *in vitro* culture and also in the growth factors, and interleukins concentrations produced during the process of cell expansion. In order to consider future clinical applications in cell therapies of the isolated hMSCs from the UCT, the use of autologous hUCBP as a culture medium supplement in the cryopreservation process of hMSCs or UCT, and *in vitro* proliferation of hMSCs, also encouraged us to know in a more detailed way the composition of the hUCBP and compare it with the CM where the hMSCs were grown and expanded by NMR spectroscopy and by Multiplexing LASER Bead Technology (Eve Technologies, Canada). Thus, knowing in detail the CM and hUCBP composition, not only in terms of growth factors and interleukins but also in terms of aminoacids, lipids and other components is important to evaluate these alternatives to hMSCs clinical application.

## Materials and Methods

### Ethics and regulation

UCB donations were obtained with written informed consents according to Directive 2004/23/EC which sets the standards of quality and safety for the donation, procurement, testing, processing, preservation, storage and distribution of human tissues and cells. The UCT and the UCB were collected from healthy donors, according to Netcord guidelines and following Portuguese law 12/2009 (Diário da República, lei 12/2009 de 26 de Março de 2009). The Biosckin, Molecular and cell Therapies SA (Maia, Portugal) is an authorized Portuguese private cord blood bank for UCB and UCT processing and cryopreservation by Direcção Geral de Saúde (DGS), and is certified for ISO9001 and for NP4457.

### Preparation of hMSCs conditioned media (CM)

Human MSCs from Wharton's jelly umbilical cord (hMSCs) were purchased from PromoCell GmbH (C-12971). Different batches of cryopreserved cells were *in vitro* cultured and maintained in a humidified atmosphere with 5% CO_2_ at 37°C. This established human MSC cell line was preferred because the number of hMSCs obtained was higher in a shorter culture time, it was not dependent on donors' availability and ethic committee authorization, and the protocol was much less time consuming, which was advantageous for the *in vitro* studies. The phenotype of hMSCs was assessed by PromoCell assay. Rigid quality control tests were performed for each lot of PromoCell hMSCs and tested for cell morphology, adherence rate and viability. Furthermore, each cell lot was characterized by flow cytometry analysis for a comprehensive panel of markers, such as platelet endothelial cell adhesion molecule – 1 (PECAM-1, CD31), homing cell adhesion molecule (HCAM, CD44), CD45, and Endoglin (CD105).

The hMSCs thawed and expanded in our laboratory exhibited a mesenchymal-like shape with a flat and polygonal morphology. The phenotype of the hMSCs expanded for the experimental work was confirmed in our laboratory by flow cytometry in order to certify that the HMSCs followed the International Society for Cellular Therapy (ISCT) criteria [9]. Detection was performed with the following antibodies and their respective isotypes (all from BioLegend unless stated otherwise): PE anti-human CD105 (eBioScience); APC anti-human CD73; PE anti-human CD90; PerCP/Cy5.5 anti-human CD45: FITC anti-human CD34; PerCP/Cy5.5 anti-human CD14; Pacific Blue anti-human CD19 and pacific-blue anti-human HLA-DR. The karyotype of undifferentiated HMSCs was determined in order to certify the absence of neoplastic characteristics in these cells, as well as the chromosomal stability to the cell culture procedures before the in vivo application [5].

Mesenchymal stem cell medium (LabClinics, PromoCell, C-28010) was replaced every 48 hours. At 80% confluence, cells were harvested with 0.25% trypsin with EDTA (Gibco) and passed into a new flask for further expansion. Two mesenchymal stem cell mediums were tested for the conditioning, namely, PromoCell medium (LabClinics, Promocell, C-28010, so called commercial medium further on) and Dulbecco's Modified Eagle Medium (DMEM, Gibco) supplemented with 10% of fetal bovine serum (FBS, Gibco), 2 mM glutamine (Sigma), 100 U/ml of penicillin and 100 µg/ml of streptomycin (Sigma). Conditioned media (CM) was collected from P4 hMSCs. To obtain the desired CM, 4000 cell/cm^2^ were plated and allowed to grow until reaching a minimum of 80% confluence. At this stage, the commercial medium was removed from the T-flasks and after 5 washing cycles with Dulbecco's Phosphate Buffered Saline 1X (DPBS) without calcium (Ca^2+^) and magnesium (Mg^2+^) (Gibco), Dulbecco's Modified Eagle Medium/Nutrient Mixture (DMEM, Gibco) supplemented with 100 U/ml of penicillin and 100 µg/ml of streptomycin (Sigma) was added. The cell culture T-flasks were maintained in a humidified atmosphere with 5% CO_2_ at 37°C, allowing the adherent cells to be in contact with a serum-free basal medium. The culture media added after the confluence was reached was not renewed, and it was collected at different time points (24 h Com. Medium and 48 h Com. Medium). For conditioning with Dulbecco's Modified Eagle Medium/Nutrient Mixture (DMEM, Gibco) supplemented with 100 U/ml of penicillin and 100 µg/ml of streptomycin (Sigma), samples of the medium were collected at 24 h (24 h DMEM) and 48 h (48 h DMEM). Upon collection, the CM were frozen at −20°C, being later on thawed on the day of the experiments. ^1^H-NMR spectra and Multiplexing LASER Bead analysis were acquired both from 24 h and 48 h samples (Table S1-S6, Figure S1).

### Preparation of umbilical cord plasma

Eight samples of hUCBP (Plasma samples #1–8) collected from different donors were used for ^1^H-NMR analysis (N = 8) and other 3 samples of hUCBP (Plasma samples #9-11) collected from different donors were tested by Multiplexing LASER Bead analysis (N = 3). The total number of hUCBP samples collected from different donors (N = 11) used in the present experimental study were analyzed by flow cytometry and for microbiological contamination for aerobic and anaerobic microorganisms and fungi.

#### Donors selection and umbilical cord blood (UCB) collection

Maternal and neonatal pairs were evaluated during the antenatal period in the maternity wards at different hospitals collaborating with the Umbilical Cord Blood Private Bank of Biosckin, Molecular and Cell Therapies, SA (Maia, Portugal). Donors signed informed consent before delivery and were clinically evaluated by the Clinical Director of the Biosckin, Molecular and Cell Therapies, SA (Biosckin bank) according to Portuguese law 12/2009 (Diário da República, lei 12/2009 de 26 de Março de 2009). UCB was collected from the umbilical vein by gravity into a 150 ml volume simple bag (reference 1385.13, Suru, Portugal) containing 21 ml of citrate-phosphate-dextrose (CPD). This procedure was performed by trained midwives after delivery of the placenta. The UCB was stored at 4°C±2°C until processing for cryopreservation. UCB samples were transported to the Biosckin SA laboratory at refrigerated temperatures ranging between 4°C and 22°C, within 48 hours after collection. The collection method was the same for all the units included in this study.

#### Volume reduction with the AXP automated system

The AXP system (Thermogenesis) consists of a microprocessor controlled device and a disposable closed blood bag set. The device contains different compartments for housing the processing set and flow control optical sensors that are used to achieve the separation of a concentrated mononuclear cell (MNC) fraction of uniform volume. The collected UCB is transferred to the bag set by means of a sterile dock tubing system, through a clot filter and loaded into the AXP device. During the two-step centrifugation, whole blood is separated into three layers that are delivered into a red blood cells (RBC) bag and a freezing bag. Plasma remains in the processing bag which is also the plasma bag. The AXP device fits most standard blood bank centrifuge buckets. In Biosckin SA laboratory, 4 UCB units were centrifuged at the same time. The programmed final volume in the cryopreservation bag was 21 ml. Samples of the UCB were taken by sampling pillows integrated within the kit for flow cytometry analysis. The analyzed UCB plasma was collected by a sterile syringe with a needle of 16G from the plasma bag.

#### Biological controls

The CD34 antigen is present on immature hematopoietic precursor cells and hematopoietic colony-forming cells in bone marrow, peripheral blood and umbilical cord blood, including unipotent and pluripotent progenitor cells. An accurate measure of the CD34^+^ cell count is necessary for dose requirements protocols on stem cell transplantation and stem cells therapies. Total nucleated cells (TNC) count, CD34^+^ cell counts, CD34^+^ cell viability and leucocytes (CD45^+^) viability were determined on samples obtained from the UCB before volume reduction. Microbiological controls were performed after volume reduction and before cryopreservation and tested for microbiological contamination using an automated blood culture system (BacT/ALERT, BioMérieux) at 35°C for 14 days. Each cord blood unit was tested for aerobic and anaerobic microorganisms and fungi using 10 ml of UCB samples consisting of mixed plasma and RBC (waste product) obtained after the AXP volume reduction procedure, which was aseptically introduced into the BacT/ALERT (BioMérieux) testing flasks. For the UCB units that presented microbial contamination, the microorganism was identified. TNC and the number of white blood cells (WBC) were counted with a hematology autoanalyser (Ac T diff2, Beckman Coulter, Inc.). The counts were not corrected for nucleated RBC. The CD34^+^ cell number and the CD34^+^ viability were quantified by flow cytometry (BD FACSCalibur 3 CA Becton Dickinson, BD Biosciences), the software for acquisition and analysis were BD CellQuest and BDCellQuest Pro Templates, respectively. The clusters of differentiation (CD) used to enumerate the total number of CD34^+^ cells and the total number of leucocytes (CD45^+^), the 7-Amino-Actinomycin D (7AAD) nucleic acid dye was used for viability measure (BD Stem Cell Enumeration kit, Becton Dickinson, BD Biosciences), according to the manufacture's protocol. The BD Stem Cell Enumeration simultaneously enumerates the total viable dual-positive (CD45^+^/CD34^+^) hematopoietic stem cells in absolute counts of CD34^+^ cells per µl and the percentage (%) of viable leucocytes (CD45^+^) that are CD34 positive (CD34^+^).

### Multiplexing LASER Bead analysis of umbilical cord blood plasma (hUCBP) and of conditioned media (CM)

Multiplexing LASER Bead Technology (Eve Technologies, Calgary, Alberta, Canada) permits the simultaneous testing of numerous analyte categories such as cytokines, chemokines and growth factors in a single assay and is based on color-coded polystyrene beads. The bead analyzer (Bio-Plex 200, Bio-Rad) includes a dual-laser system and a flow-cytometry system. One laser activates the fluorescent dye within the beads which identifies the specific analyte. The second laser excites the fluorescent conjugate (streptavidin-phycoerythrin) that has been bound to the beads during the assay. The amount of the conjugate detected by the analyzer is in direct proportion to the amount of the target analyte. The results are quantified according to a standard curve using 25 µl of hUCBP, unconditioned culture medium and CM. The measurements were performed by Eve Technologies, Calgary, Alberta, Canada, according to the indications of the company. Human Primary Cytokine Array/Chemokine Array 41-Plex Panel (Eve Technologies, Calgary, Alberta, Canada) was performed to analyze the UCB plasma and the several conditioned media, including the following cytokines, chemokines and growth factors: epidermal growth factor (EGF), eotaxin-1, fibroblast growth factor 2 (FGF-2), fms-related tyrosine kinase 3 ligand (Flt-3L), fractalkine, granulocyte colony-stimulating factor (G-CSF), granulocyte macrophage colony-stimulating factor (GM-CSF), GRO(pan), interferon- alpha 2 (IFNα2), interferon-gama (IFNγ), several interleukins (IL-1α, IL-1β, IL-1ra, IL-2, IL-3, IL-4, IL-5, IL-6, IL-7, IL-8, IL-9, IL-10, IL-12 (p40), IL-12 (p70), IL-13, IL-15, IL-17A), interferon gama-induced protein 10 (IP-10), monocyte chemotactic protein-1 (MCP-1), monocyte chemotactic protein-3 (MCP-3), macrophage-derived chemokine (MDC), macrophage inflammatory protein- 1 alpha (MIP-1α), macrophage inflammatory protein- 1 beta (MIP-1β), platelet-derived growth factor – AA (PDGF-AA),), platelet-derived growth factor – AB/BB (PDGF-AB/BB), chemokin (C-C motif) ligand 5 (RANTES or CCL5), soluble CD40 ligand (sCD40L), transforming growth factor alpha (TGFα), tumor necrosis factor alpha (TNFα), tumor necrosis factor beta TNFβ, vascular endothelial growth factor A (VEGF-A). TGF-β 3-Plex Array Multi-Species (Eve Technologies, Calgary, Alberta, Canada) was also performed to analyze the UCB plasma and the several conditioned media, including tumor growth factor beta 1, 2 and 3 (TGF-β 1, 2, and 3). The samples were kept at −20°C until the analysis was performed (Table S1-S6 and Figure S1).

### NMR spectroscopy

A 600 µL aliquot of the supernatant for each tested sample was transferred into 5 mm NMR tubes and mixed with 50 µL deuterium oxide (D_2_O) containing 0.05 mM sodium trimethylsilyl-[2,2,3,3-d4]-propionate (TSP) as an internal reference for the calibration and quantification of NMR spectra. The NMR experiments were recorded at 300K on a Bruker Avance III 600 HD spectrometer, operating at 600.13 MHz for ^1^H, equipped with 5 mm CryoProbe Prodigy and pulse gradient units, capable of producing magnetic field pulsed gradients in the z-direction of 50 G/cm. Standard or slightly modified Bruker library pulse programs were used for all NMR experiments. ^1^H-NMR spectra of the samples studied were acquired with water suppression using a 1D NOESY (noesygppr1d) pulse sequence (recycle delay-90°-t1-90°-tm- acquire) and a Carr-Purcell-Meiboom-Gill (CPMG, cpmgpr1d) pulse sequence (recycle delay-90°-[τ-180°-τ]n- acquire) [26], [34], [35]. The 1D NOESY spectra were collected using 5s relaxation delay, t1 delay of 4 µs and mixing time (tm) of 0.01 s. A water pre-saturation pulse was applied throughout the relaxation delay and mixing time. The 1D NOESY experiment generates spectra with improved solvent peak (at 4.70 ppm) suppression. Relaxation edited ^1^H-NMR spectra with T2 (spin-spin relaxation time) filter using CPMG pulse sequence and suppression of water resonance were acquired to facilitate the identification of low molecular weight metabolites, reducing signals from high molecular weight species or systems in intermediate chemical exchange. The CPMG experiments (cpmgpr1d) were acquired with relaxation delay 4s, during which the water resonance signal was selectively irradiated, the echo time (τ) was optimized for each sample and was between 0.3 and 0.8 ms, and a loop for T2 filter (n) 20 was used. For all ^1^H-NMR spectra 128 or 256 transients of a spectral width of 10000 Hz were collected into 32 K time domain points. The NMR spectra were processed conventionally using TOPSPIN 3.2 (Bruker Biospin) and referenced to the TSP signal (δ = 0.00). The time domain data were multiplied by an exponential function with line-broadening factor of 0.3 Hz and zero-filled to 64 k prior to Fourier transformation. Additionally, to confirm the chemical shift assignment of ^1^H-NMR spectra and facilitate the examination of metabolites presented in biofluids studied2D ^1^H/^1^H COSY, ^1^H/^1^H TOCSY and ^1^H/^13^C HSQC spectra were acquired Typical conditions for the ^1^H/^1^H COSY and TOCSY spectra recorded in phase sensitive mode and with water suppression were: relaxation delay 2s, 16 or 32 scans, a total 2K data points in F2 and 256 or 512 data points in F1 over a spectral width of 10000 Hz. The TOCSY spectra were recorded by use of the MLEV-17 spin lock sequence [36] for proton-proton transfer with a mixing time of 80 ms. ^1^H/^13^C HSQC experiments (Bruker pulse program hsqcedetgpsisp2.2) [37]–[40] recorded in phase sensitive mode using echo/antiecho-TPPI gradient selection, with decoupling during acquisition time and multiplicity editing during selection were carried out with a spectral width of 10000 Hz for ^1^H and 27000 Hz for ^13^C, relaxation delay 1.5 s, Fourier transform (FT) size 2K×1K.

The metabolite content into the samples studied was defined by quantitative NMR analysis, using the relative quantitative method [41]. The quantitative distribution of NMR-visible metabolites was determined from the relative integral intensity of characteristic signals in ^1^H-NMR spectra of the samples referenced to the integral intensity of TSP (internal standard), considering the number of the contributing nuclei for that particular resonance (Table S1-S6, Figure S1).

### Statistical analysis

When relevant, data is presented as mean and standard deviation (SD), N is referred to the number of samples analyzed or used in the study. Statistical comparisons of quantitative data were subjected to one-way ANOVA test. All statistical procedures were performed by using the statistical package SPSS (version 14.0, SPSS, Inc). Statistical significance was accepted at p<0.05.

## Results

### Flow cytometry and microbiological analysis of the umbilical cord blood (UCB) units

Table 1 resumes the data obtained by flow cytometry which includes total number of viable CD34^+^ cells per µl, the percentage (%) of viable leucocytes (CD45^+^) that are CD34^+^, total number of viable leucocytes (CD45^+^) per liter, and the % of viable leucocytes (CD45^+^), and by hematology autoanalyser which includes the TNC per liter. The samples presented an average of 28.96±17.57×10^9^ TNC per liter, of 61.77±53.94 viable CD34^+^ cells per µl, 89.90±10.78% of CD34^+^ viability, 18.65±12.67×10^9^ viable leucocytes (CD45^+^) per liter, and 89.40±8.54% of leucocytes (CD45^+^) viability (N = 11), demonstrating individual variability considering the UCB hematopoietic stem cells concentration and CD45^+^ CD34^+^ cell viability.

**Table 1:** Total nucleated cells per liter of umbilical cord blood collected measured (TNC cell x 10^9^/l) obtained by using the hematology autoanalyser (Ac T diff2, Beckman Coulter, Inc.), number of viable CD34^+^ cells per µl of UCB collected (cells/µl), viability of CD34^+^ cells (%), number of viable CD45^+^ cells per liter of UCB collected (cells ×10^9^/l) and viability of CD45^+^ cells (%) measured by flow citometry of the UCB samples used for ^1^H NMR and Multiplexing LASER Bead analysis.

	TNC (cell x 10^9^/l)*	Viable CD34+ (cells/µl)	Viability of CD34+ (%)	Viable CD45+ (cells x 10^9^/l)	Viability of CD45+ (%)
**Plasma sample #1**	54.30	73.00	98.20	34.00	96.00
**Plasma sample #2**	66.00	182.00	99.74	47.30	96.37
**Plasma sample #3**	26.60	28.00	86.70	17.10	92.30
**Plasma sample #4**	10.20	11.40	75.70	5.70	73.00
**Plasma sample #5**	19.90	26.00	83.60	14.30	83.70
**Plasma sample #6**	22.50	21.10	66.10	18.00	94.50
**Plasma sample #7**	24.10	60.00	96.60	10.60	88.00
**Plasma sample #8**	24.70	35.00	94.44	20.00	96.68
**Plasma sample #9**	17.20	77.00	97.10	7.70	75.50
**Plasma sample #10**	40.70	138.00	94.50	23.90	96.50
**Plasma sample #11**	12.40	28.00	96.20	6.60	90.80
**Mean**	28.96	61.77	89.90	18.65	89.40
**SD**	17.57	53.94	10.78	12.67	8.54

The average and standard deviation (SD) of N  =  11 is also presented. * Count using the hematology autoanalyser.

Considering the microbiological evaluation of the UCB plasma performed using an automated blood culture system (BacT/ALERT, BioMérieux) at 35°C for 14 days as it was described previously, all the samples were negative for aerobic, anaerobic microorganisms and fungi except plasma sample #2 which was positive for *Bacteroides distasonis* in anaerobiosis. The bacterial contamination did not interfere with ^1^H-NMR analysis and no statistical differences were observed between plasma sample #2 and the mean values calculated for the 8 plasma samples analysed (N = 8, p<0.05) (Table S2).

### Multiplexing LASER Bead analysis of umbilical cord blood plasma (hUCBP), unconditioned and conditioned media (CM)

**Figure 1:**
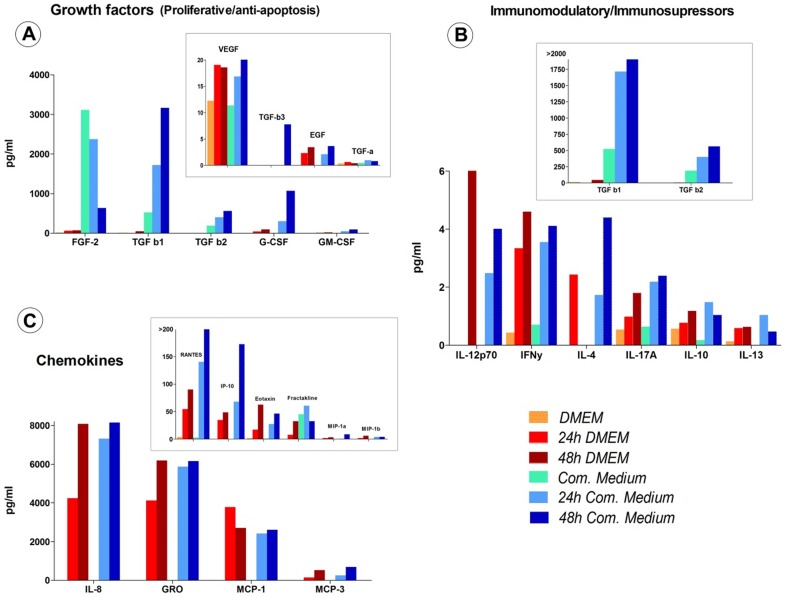
Proliferative and anti-apoptotic growth factors (A), immunomodulatory, immunosupressive cytokines (B) and chemokines (C) concentrations in unconditioned (Com. Medium* and *DMEM*) and conditioned media (*24/48 h Com. Medium* and *DMEM*). (Multiplexing LASER Bead Analysis (Eve Technologies, Calgary, Alberta, Canada). *Commercial medium from PromoCell (LabClinics, Promocell, reference C-28010).

**Figure 2:**
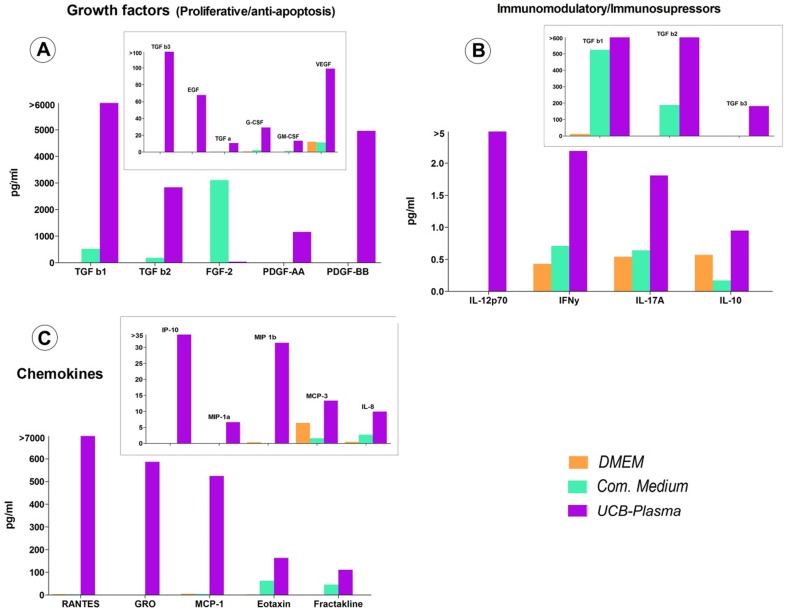
Proliferative and anti-apoptotic growth factors (A), immunomodulatory, immunosupressive cytokines (B) and chemokines (C) concentrations in *UCB Plasma* and unconditioned media (Com. Medium* and *DMEM*). (Multiplexing LASER Bead Analysis (Eve Technologies, Calgary, Alberta, Canada). *Commercial medium from PromoCell (LabClinics, Promocell, C-28010).

The results gathered from the Human Primary Cytokine Array/Chemokine Array 41-Plex Panel together with the TGF-β 3-Plex Array, allowed us to perform a comprehensive analysis of the cytokines and growth factors included in the hUCBP and CM in comparison with both DMEM and a commercial culture media. In terms of the proliferative and anti-apoptotic factors it was clear that the conditioned samples (both with DMEM and *Com. Medium*) presented an increase in terms of concentration of these factors. This was particularly evident for TGF-β1 (24 h and 48 h *Com. Medium*), EGF, G-CSF, GM-CSF, PDGF-AA and VEGF. Some of these factors like EGF were even absent in unconditioned media. It must be also stated that factors such as TGF-β1 and G-CSF were present in considerable increased concentrations in 48 h conditioning samples (Figure 1 and Figure 2). TGF-β1 is associated with stem cell differentiation and protection and notably for its anti-apoptotic effects. G-CSF is known for progenitor cells mobilization and also for its anti-apoptotic effects (revised by Doorn et al. [3]).

hUCBP presented very high concentrations of TGFβ(1-3) that were completely out of the range found in both conditioned and unconditioned culture media. For example TGF-β1 had a 5-fold lower concentration in the 48 h *Com. Medium* compared to the hUCBP (3162 pg/ml VS 16670 pg/ml). This was also observed in lower scale, with factors like VEGF, PDGF-AA, PDGF-BB and EGF. The tendency observed for the increased values in conditioned (vs unconditioned) samples was also observed in all tested chemokines and mostly in MCP-1, MCP-3, RANTES, GRO, IL-8. It should be noticed that in hUCBP, RANTES was detected with a concentration of 78903 pg/ml, while other samples ranged from 2.863 to 252 pg/ml. hUCBP also presented higher concentrations of eotaxin and fractakline compared to the culture media analyzed (163 pg/ml VS 1,24-62 pg/ml and 110 pg/ml VS 0-60 pg/ml respectively). Apart from the already referred TGF family, other immunossupressive/immunomodulatory factors were predominantly high in the conditioned and hUCBP (Figure 1 and Figure 2, Table S1 and S3).

### ^1^H-NMR analysis of the umbilical cord blood plasma (hUCBP) and conditioned media (CM)

The identification of the metabolites in the samples studied was achieved by analysis of the high resolution ^1^H-NMR spectra, considering NMR parameters such as chemical shifts, multiplicity and intensity, characteristic for the respective species. The unambiguous assignment of the resonance signals in the ^1^H-NMR spectra was achieved by verification of the results through 2D NMR (^1^H/^1^H COSY, ^1^H/^1^H TOCSY, ^1^H/^13^C HSQC) spectroscopy. The assignment of the resonance signals of the specific metabolites in ^1^H-NMR spectra of the samples studied were in agreement with the data published in the literature [42], [43].

**Figure 3:**
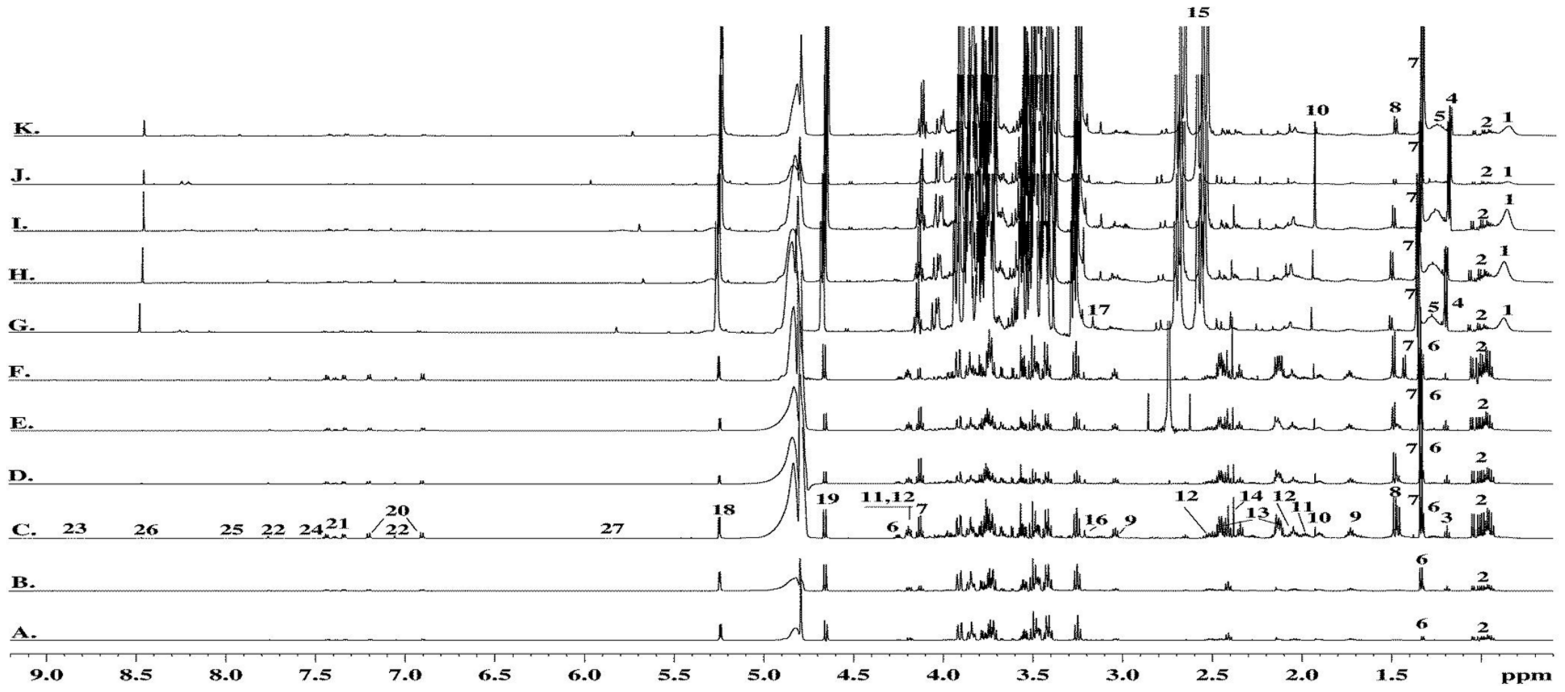
600 MHz ^1^H-NMR spectra of: A. DMEM; B. 24 h DMEM; C. 48 H *Com. Medium*; D. 24 h *Com. Medium* ^1^; E. 24 h *Com. Medium* ^2^; F. *Com. Medium*; G. *Plasma1*; H. *Plasma2*; I. *Plasma3*; J. *Plasma4*; K. *Plasma5*. The codes (1-28) represent metabolites from **Table S5**. *D and E correspond to two different samples with the same basal medium (*Com. Medium*) and time of conditioning (24 h). *Com. Medium is* Commercial medium from PromoCell (LabClinics, Promocell, C-28010).

**Figure 4:**
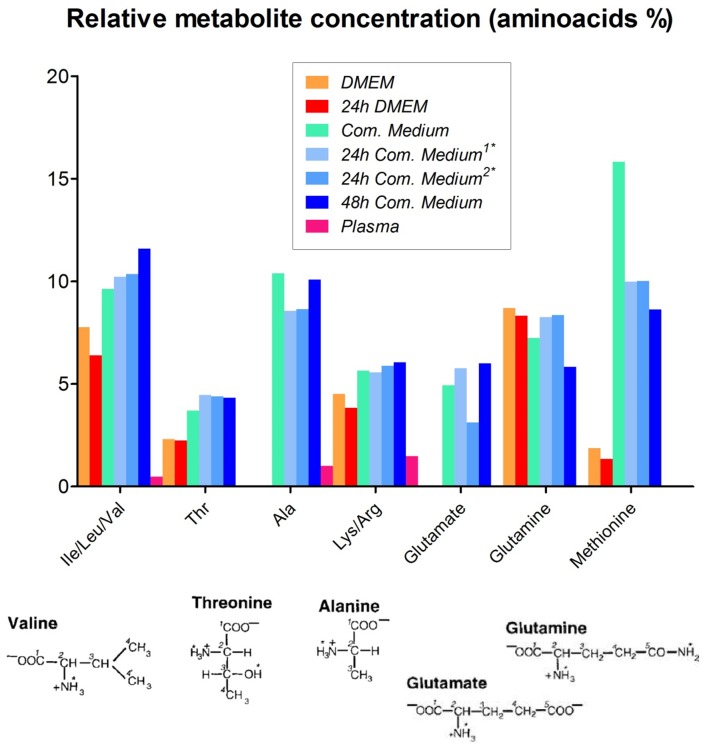
Graphical representation of the relative metabolite concentration of the main aminoacids after normalization of the integral signal intensity obtained in the ^1^H-NMR spectra of *DMEM*, *24 h DMEM*, *Com. Medium*, *24 h* and *48 h Com.* *Medium* and *UCB Plasma* (average) samples. Chemical structures of the main metabolites are represented. *Com. Medium is* Commercial medium from PromoCell (LabClinics, Promocell, C-28010).

**Figure 5:**
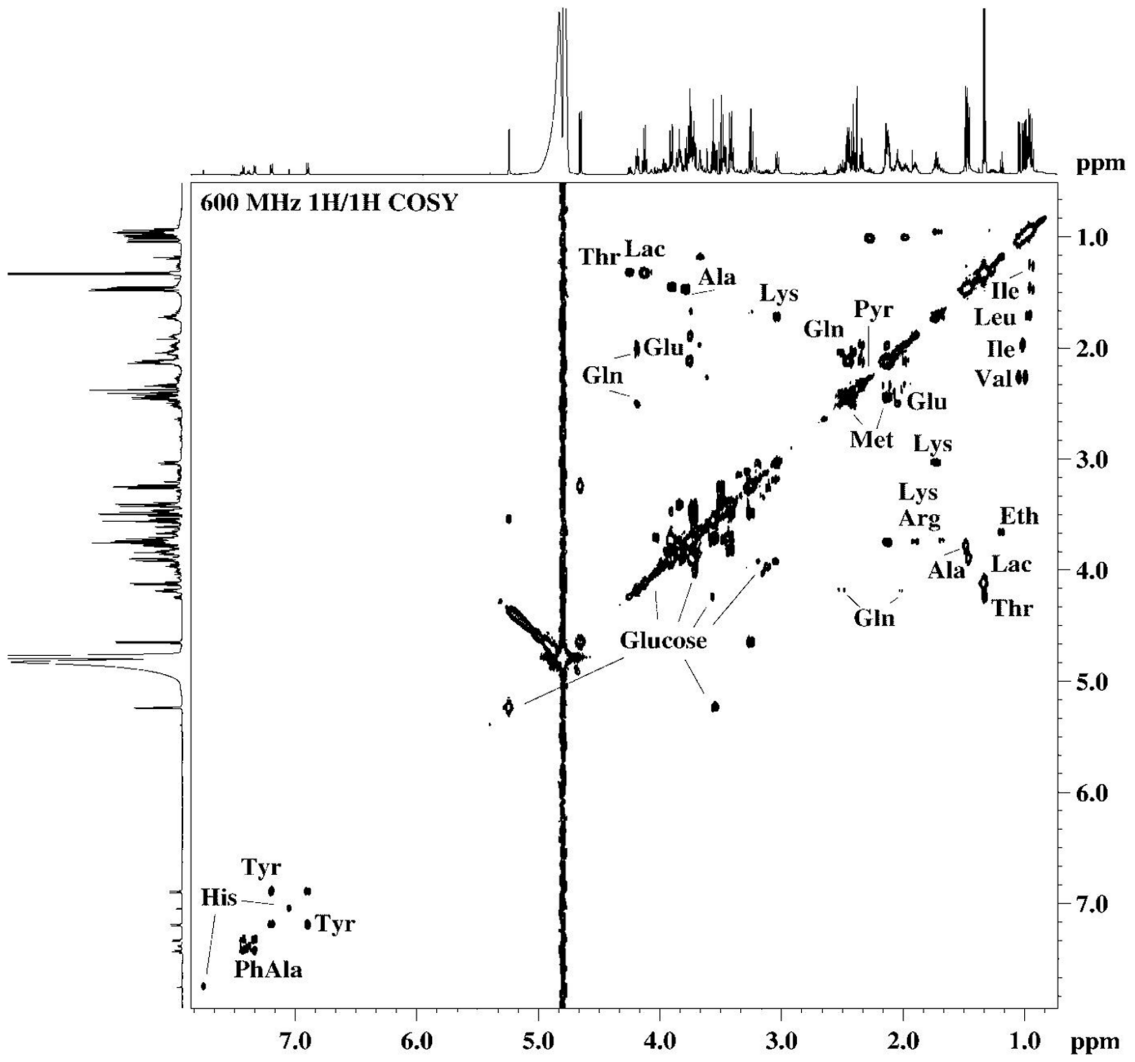
600 MHz ^1^H/^1^H 2D COSY NMR spectra of *48 h Com. Medium* sample. *Com. Medium is* Commercial medium from PromoCell (LabClinics, Promocell, C-28010).

**Figure 6:**
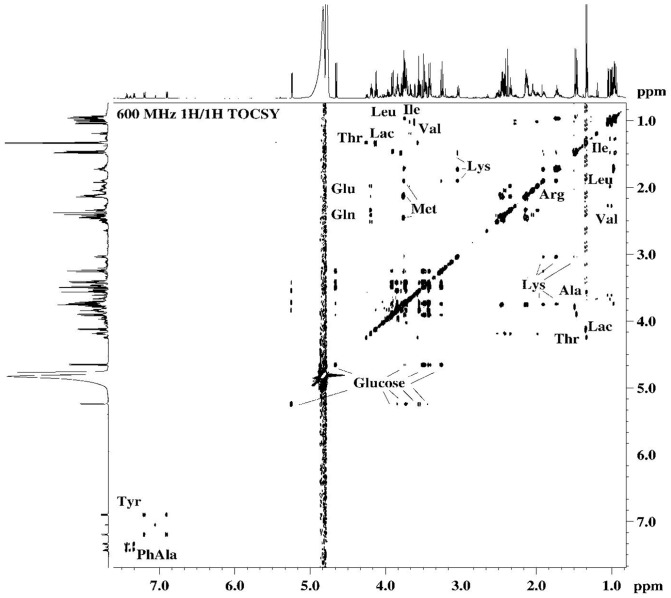
600 MHz ^1^H/^1^H 2D TOCSY NMR spectra of *48 h Com. Medium* sample. *Com. Medium is* Commercial medium from PromoCell (LabClinics, Promocell, C-28010).

**Figure 7:**
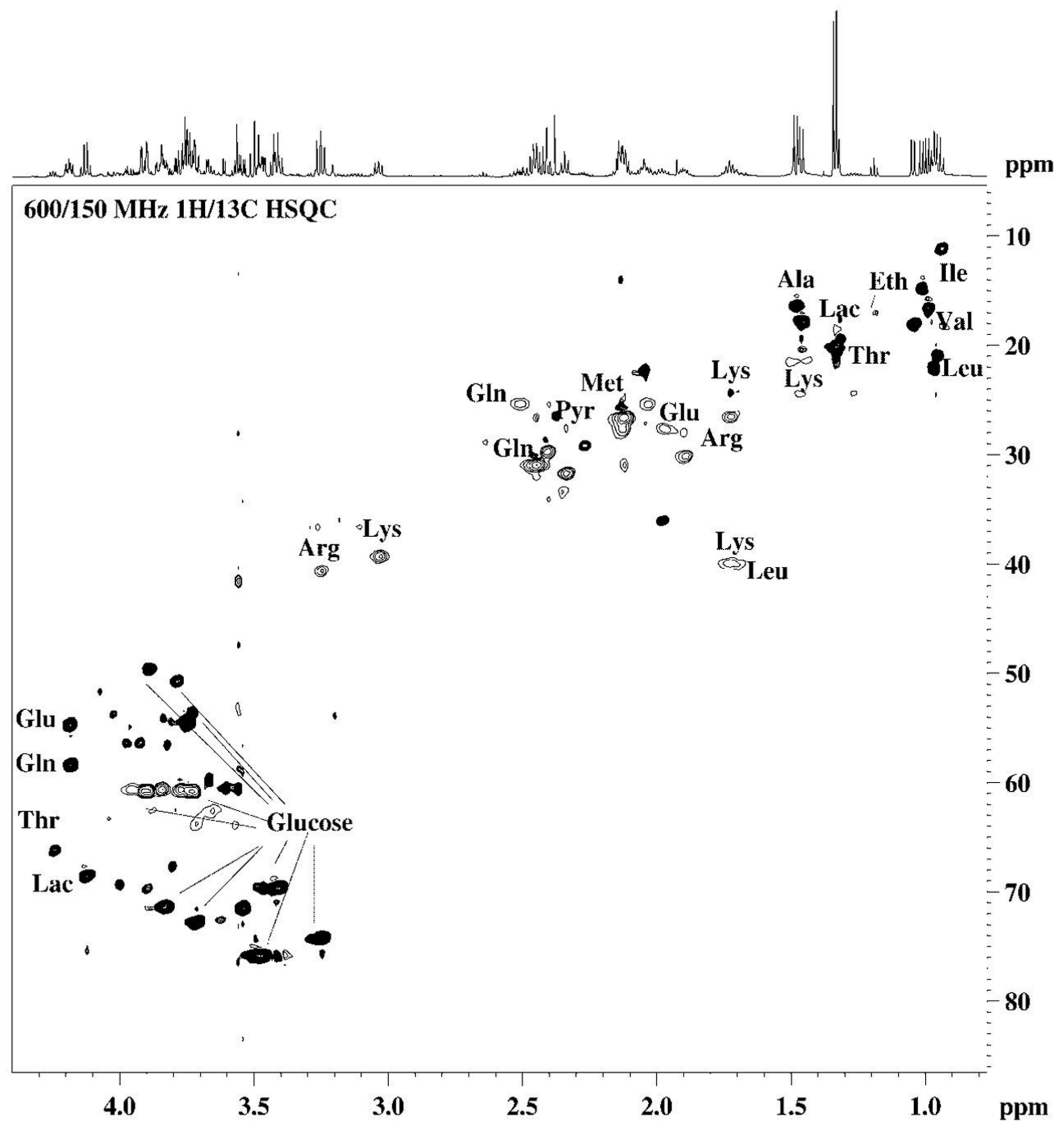
600/150 MHz ^1^H/^13^C 2D HSQC NMR spectra of *48 h Com. Medium* sample. *Com. Medium is* Commercial medium from PromoCell (LabClinics, Promocell, C-28010).

The spectral features corresponding to the main metabolites detected in the ^1^H-NMR profiles (Figure 3-7) of the different samples were transposed to Table 2 (and Table S2 and S4). In the hUCBP samples the citric acid value presented high values in terms of the integrals for signal intensity because this metabolite was originated from the anticoagulant used in sample collection rather than being present in the plasma. In the basal culture media (DMEM and *Com. Medium*) as well as the corresponding conditioned media (24 h DMEM, 24 h *Com. Medium* and 48 h *Com. Medium*) a number of important amino acids, glucose and low molecular weight (lactate, acetate, pyruvate, formate) compounds were detected through ^1^H-NMR. The most significant in terms of relative quantity were highlighted as a graphical representation in Figure 4. Alanine and glutamate were absent in DMEM and 24 h DMEM but were clearly perceived in the *Com. Medium* and 24 h/48 h *Com. Medium* samples. In a minor scale this was equally valid for succinate. Lactate was also absent in DMEM but it could be detected in 24 h DMEM. Nevertheless both in *Com. Medium* and mostly in 24 h/48 h *Com. Medium*, a significant higher concentration of lactate was registered. Other major differences between cell culture media were found in the methionine content, appearing to be present in larger amounts in the *Com. Medium* and corresponding CM (24 h and 48 h). Higher levels of glucose were detected in DMEM and 24 h DMEM comparing to *Com. Medium* and 24 h/48 h Com. Medium levels. Obviously there was also a reduction in glucose in the CM in which the basal media (DMEM and *Com. Medium*) was in contact with the hMSCs. Gathering the results of unconditioned media (DMEM and *Com. Medium*) and comparing them globally with the different CM it is possible to attest not only to metabolite depletion through cell metabolization (as the glucose example), but also some metabolites (like lactate, formate or pyruvate) that were present in higher concentration, reflecting the production and secretion of metabolites. Metabolic differences between samples collected at different time points (24 h and 48 h) were also considered. This is because CM could be achieved at different stages after reaching minimal cell confluence. Comparing (24 h) to (48 h) conditioning with *Com. Medium*, the major differences could be detected in terms of a decrease in acetate and lactate together with an increase in alanine values. In terms of the average results for the hUCBP samples and in contrast with the culture and conditioned media, it was possible to detect lipids, scyllo-inositol, β-hidroxibutirate and purines. However, we were unable to identify signals for some aminoacids like threonine, glutamate, glutamine, methionine and other metabolites like thiamine, choline or nicotinamide. Considering the different hUCBP analyzed (Table S2, Figure 3, and Figure 4) and comparing with the results obtained with CM (Table S2, Figure 3-7) only differences in the lipids, β-hydroxybutyrate (β-HB), and inositol content are significant. As a matter of fact these components are present in the 8 samples of hUCBP analyzed and are absent in the CM of the *in vitro* expanded hMSCs. In addition, the significantly higher intensity of the resonance signals of α-glucose and β-glucose in 1H- NMR spectra of hUCBP compared with the CM reveals the significantly higher concentration of α- and β-glucose in hUCBP. This is consistent with the function of the UCB to provide energetic support for the fetus during intrauterine development. It is also due to the presence of anticoagulant CPD in the UCB collecting bag. The UCB collecting bag of the UCB has CPD as an anticoagulant with the following composition: 2.63 g of sodium citrate (dehydrate), 0.299 g of citric acid (monohydrate), 2.55 g of dextrose (monohydrate) and 0.222 g of sodium monobasic biphosphate (monohydrate). It is also important to keep in mind the great variability of the UCB samples collected in terms of hematopoietic stem cells (CD34^+^ cells) (Table 1) and hMSCs concentration which may influence the composition of the hUCBP considering the different components analyzed by ^1^H-NMR. As matter of fact, sample#4 presented a very low concentration of hematopoietic stem cells CD34^+^ (10.2 viable CD34^+^ cells/µl) which might explain the different results obtained by ^1^H-NMR when compared to the other samples of hUCBP (Table 1 and Table S2).

**Table 2:** Proliferative and anti-apoptotic growth factors (**A**), immunomodulatory, immunosupressive cytokines (**B**) and chemokines (**C**) concentrations in *UCB Plasma*, unconditioned (*Com. Medium* and *DMEM*) and conditioned media (*24/48 h Com. Medium* and *DMEM*).

Code	Metabolites	Group	Chemical shifts (ppm)	DMEM	24 h DMEM	Com. Medium	24 h Com. Medium	48 h Com. Medium	Plasma (average)
**1**	Lipids	CH_3_	0.87	-	-	-	-	-	2.46
**2**	Ile/Leu/Val	CH_3_	0.9-1.0	1.58	1.85	4.30	2.96	4.07	0.49
**3**	Ethanol	CH_3_	1.18	-	0.42	0.15	0.51	0.47	-
**4**	β-HB	CH_3_	1.19	-	-	-	-	-	1.93
**5**	Lipids	CH_2_	1.28	-	-	-	-	-	3.62
**6**	Threonine	CH_3_	1.33	0.47	0.65	1.65	1.275	1.52	-
**7**	Lactate	CH_3_	1.34	-	2.64	3.05	6.24	5.09	7.41
**8**	Alanine	CH_3_	1.48	-	-	4.64	2.475	3.54	1.00
**9**	Lys/Arg	CH/CH_2_	1.6-1.8	0.92	1.11	2.52	1.645	2.13	1.47
**10**	Acetate	CH_3_	1.92	0.14	0.14	0.47	0.3	0.24	0.56
**11**	Glutamate	CH_2_	2.36	-	-	2.20	1.295	2.11	n.d.*
**12**	Glutamine	CH_2_	2.51	1.77	2.41	3.23	2.39	2.05	n.d.*
**13**	Methionine	CH_2_	2.45	0.38	0.39	7.06	2.88	3.03	n.d.*
**14**	Piruvate	CH_2_	2.39	-	-	1.19	0.455	0.67	0.24
**15**	Citric acid	CH_2_	2.62	-	-	-	-	-	92.69
**16**	Choline	NCH_3_	3.21	-	0.01	0.09	0.065	0.09	n.d.*
**17**	Inositol	CH	3.37	-	-	-	-	-	1.67
**18**	α-Glucose	H1	5.24	7.11	8.19	5.08	2.27	3.73	33.26
**19**	β-Glucose	H1	4.65	7.10	10.07	6.75	2.685	4.52	42.70
**20**	Tyrosine	H3.5	6.90	0.31	0.37	0.79	0.455	0.63	0.11
**21**	Phenylalanine	H4	-	0.30	0.38	0.85	0.455	0.66	0.14
**22**	Histidine	H5	7.05	0.16	0.17	0.42	0.235	0.30	0.16
**23**	Nicotinamide	H2	8.89	0.03	0.03	0.06	0.04	0.05	n.d.*
**24**	Tryptophan	H4	7.74	0.06	0.06	0.07	0.055	0.06	0.06
**25**	Thiamine	H12	8.03	0.01	0.01	0.01	0.01	0.01	n.d.*
**26**	Formate	CH	8.47	0.01	0.03	0.06	0.05	0.06	1.13
**27**	Urea	NH_2_	5.90	-	-	-	0.04	0.09	0.12
**28**	Purines	CH	8.1-8.4	-	-	-	-	-	0.70

(Multiplexing LASER Bead Analysis (Eve Technologies, Calgary, Alberta, Canada). – <10 pg/ml; ± ≧10 <100 pg/ml; + ≧100 <1000 pg/ml; ++ ≧1000 <2000 pg/ml; +++ ≧2000 <3000 pg/ml; ++++ ≧3000 <4000 pg/ml; +++++ ≧4000 <5000 pg/ml; ++++++ ≧5000 pg/ml; **O** Out of Test Range.

## Discussion

hMSCs represent an appealing source of adult stem cells for cell therapy and tissue engineering. Because hMSCs are present in low percentage in the bone marrow, alternative sources have been studied, like the umbilical cord tissue (UCT, also called Wharton's jelly). In addition, *in vitro* expansion is necessary before performing clinical studies. The hMSCs are capable of differentiation along osteogenic, chondrogenic and adipogenic lineages [44], [45]. Recent *in vivo* and *in vitro* studies also have demonstrated the capacity to repair and regenerate cartilage [46], bone, tendon, and meniscus [47]–[49]. The hMSCs are also able to differentiate into cardiomyocytes [50], [51], skeletal muscle and neuro-glial cells [23], [52] which has also been intensively studied *in vitro* and *in vivo* using animal models, by our research group. The hMSCs have been used in several clinical trials in children and adults [11] over a wide range of pathologies and diseases. hMSCs have also been intensively studied to promote engraftment in allogenic hematopoietic stem cell transplantation. In fact, hMSCs, particularly the ones isolated from the Wharton's jelly, are nowadays used as a co-adjuvant in hematopoietic treatments using UCB and bone marrow transplantation [53], [54]. Nowadays, the cryopreservation of UCB and UCT is performed worldwide in private and public cord blood banks because the umbilical cord blood is used in hematopoietic treatments for blood disorders and hemato-oncological diseases. Furthermore, the co-transplantation of hMSCs and hematopoietic stem cells have positive clinical outcomes in hematologic malignancy patients [55]. For instance, the therapeutic dosage of hMSCs commonly employed for infusion in an adult patient for treatment of graft-*versus*-host disease in higher than 2×10^6^ cells/kg body weight. For this reason the *ex vivo* expansion of hMSCs for therapeutic applications concerning cell therapies is necessary in almost every clinical case [56]. Human MSCs can influence tissue regeneration and scar tissue formation processes mainly by their paracrine effect through a range of biomolecules synthesized by these cells, more than their direct differentiation into functional tissue. The niche created by the expression of chemotactic factors such as Wnt3a, VEGF, and PDGF attracts endogenous hMSCs to the area of injury [1]. Although transplanted hMSCs do not persist well in the graft environment [57], they are able to initiate the formation of this niche in the injured environment promoting the mobilization of endogenous stem/progenitor cells to the site of injury. In their work, Shin and Peterson [1] observed that the engrafted cells disappeared rapidly [being susceptible to death by pro-inflammatory cytokines and reactive oxygen species [57]] and at that moment there was a correspondent increase in the number of host cells to the area of injury. They also perceived the modulation and recruitment of host cells both locally and systemically in response to exogenous MSC engraftment [1]. These transplanted hMSCs also have the value of acting as “protectors” to other cell types [2]. Understanding the role of the various mechanisms involved in the environment of these stem cell “niches” is extremely important to understanding not only the concept of stem cell biology but also for the establishment of *in vitro* culture protocols meant for biomedical use [6]. The detailed characterization of hMSCs secretome is becoming particularly relevant because the factors secreted by these cells may be the main effectors of their therapeutic action [6]. The low survival rate of transplanted cells into the damaged myocardium has also been proved [58]. Consequently, the clinical benefits are only transient and attributed mostly to transplanted cell-associated paracrine effects that for example stimulate angiogenesis by stimulating endothelial cell adhesion through chemotactic factors [58], [59]. This recent paradigm has suggested that the biomolecules synthesized by stem cells may be as important, if not more so, than differentiation of the cells in eliciting functional tissue repair [2]. This evidence suggests that CM obtained from the *in vitro* culture and expansion of hMSCs and hematopoietic stem cells (CD34^+^ cells) or hUCBP are probably better therapeutic options compared to the *in vivo* transplantation of these stem cells. This is because the regenerating tissues can benefit from the local tissue response to the secreted molecules without the difficulties and complications associated to the engraftment of the allo-transplanted or xeno-transplanted cells [5]. Apart from that, stem and progenitor cells have been subjected to *in vitro* preconditioning regimens to increase production of desired trophic factors (as with hypoxia and angiogenic factor production), thereby augmenting cell paracrine actions [2]. CM derived from hMSCs was demonstrated to contain factors that promote recruitment of macrophages and endothelial cells into the wound [60]. Apart from hMSCs, CM alone also has substantial effects on migration, proliferation, and overall wound [3]. For example Li et al. [61] demonstrated that hMSCs CM can directly inhibit proliferation of cardiac fibroblasts and gene expression of collagen I and III. In addition to immunoregulatory, pro-angiogenic, and anti-apoptotic factors, hMSCs also secrete neurotrophic factors, which could potentially be used in neurological disorders [3], [62]–[64]. Previously, the CM from expanded hMSCs was *in vivo* tested and the therapeutic effect was compared to hMSCs local application associated to different vehicles, in the rat skeletal muscle myectomy lesion model with positive clinical outcomes [5]. These results might be explained by the fact that proliferation of satellite cells (SCs) is regulated by FGFs, TGF-βs, PDGF, IGF-I and II, while differentiation appears to be promoted mainly by IGFs [65] and as we demonstrated in this study, some of these factors that are important in skeletal muscle regeneration are present in hMSCs from the Wharton's jelly conditioned media. The results obtained from the Multiplexing LASER Bead analysis for the CM (using both DMEM and *Com. Medium*) revealed a higher concentration of all the main cytokines when compared to the corresponding unconditioned media. The only exception was FGF-2 which is an anti-apoptotic, anti-fibrotic growth factor with an important role in increasing proliferation (and repressing differentiation) [3], [57], [65]. FGF-2 appeared in higher concentrations in 24 h and 48 h DMEM (CM) compared to unconditioned DMEM. However the unconditioned commercial medium used in this study showed high content in FGF-2 (3111 pg/ml), which was even higher than the corresponding CM (24 h and 48 h *Com. Medium*) (Table S1). FGF-2 might be included in the unknown composition of the supplement cocktail used in the commercial medium. In fact this is also consistent with the better *in vitro* performance of this commercial medium in terms of hMSCs proliferation in culture. FGF and other growth factors like VEGF and MCP-1 (that were also increased in our CM samples) are known to be cardioprotective. These results are important regarding the conclusions presented in Zisa et al. [66], in which the beneficial effects of CM on heart function after myocardial infarcts was attributed to such soluble factors. Also increased in our CM analysis (Table S1 and Figure 1); IL-6, IL-8, and MCP-1 have been demonstrated to enhance migration of monocytes, which suggests enhanced attraction of these cells to the site of injury *in vivo* [3]. Cytokines such as IL-10 and TGF-β which in our data can be identified in higher concentration in CM (compared to unconditioned media), have anti-proliferative effects on T-cells and decrease the secretion of inflammatory proteins, such as TNF-α and IFN-γ [3]. The TGF-β family apart from being anti-apoptotic, has an important function in stem cell protection and differentiation (particularly TGF-β3) [3], [57].Also anti-apoptotic, G-CSF and GM-CSF are widely exploited clinically to mobilize stem cells into the peripheral blood [3]. Caplan and Dennis [7] have also witnessed that G-CSF promoted the marrow hMSCs' migration into the heart after myocardium infarction. In the present results, the concentration of both factors was clearly raised in CM (comparing to unconditioned media) and was even higher in the hUCBP.

On the basis of the results obtained from the analysis of the 1D and 2D NMR spectra acquired and by comparison with literature values, we manage to define the NMR-visible metabolites presented in the samples studied (Figure 1-7; Table S2-S5). The ^1^H-NMR spectra of the samples studied were acquired with water suppression using a 1D NOESY and CPMG pulse sequences. Using CPMG sequences, we achieved better suppression of the water resonance, clearer inspection of the signals arising from small molecules such as amino acids, lactate etc., and reduction of signals from high molecular weight species or systems in intermediate chemical exchange. However, the lipid signals could be attenuated at the expense of the enhancement of the resonance signals of low molecular weight metabolites. The NMR spectral data we obtained are in agreement with the data published in the literature [42], [43], [67]. In the results obtained from the different CM, the differences detected (compared to unconditioned media) were basically a consequence of cell metabolization (as the glucose depletion for example). Lactate, formate and pyruvate were present in higher concentration in CM, reflecting the production and secretion of metabolites. The presence of glutamate, pyruvate and alanine only in the commercial medium and corresponding CM (24 h and 48 h *Com. Medium*) might be explained by the use of supplements such as Glutamax in this commercial medium. When hydrolyzed, this supplement produces higher levels of alanine [33]. Glutamine is also a product of this process, but the utilization of this aminoacid by hMSCs should also be considered. Apart from pyruvate, acetate levels may also be influenced by the freezing or thawing process [33].

Nowadays the *in vitro* protocols for hMSCs expansion include the FBS as a supplement of the culture medium and currently there are no reports of any significant side effects due to the presence of xenogenic proteins. As a matter of fact, in phase I clinical trials has been authorized but later-phase studies and clinical therapies would strictly require serum-free or xeno-free culture media. Although the posterior sequential culture of the hMSCs in autologous or heterologous plasma can remove up to 99.99% of the xenogenic proteins, a residual risk still remains, including the possibility of prions and virus transmission [11]. The selection of an alternative for the FBS is crucial, and one important possibility is the UCB plasma or serum. The analysis of the hUCBP through ^1^H-NMR and Multiplexing LASER Bead would aid in the identification of specific components and factors that have *in vitro* promoting effects on hMSCs expansion. This will help in defining serum-free culture medium for hMSCs expansion. Also, the autologous or heterologous hUCBP is a cheaper alternative for the large-scale expansion of hMSCs. At the moment no definitive FBS serum free and chemically defined culture medium with all the important growth factors is available in the market for the hMSCs. However, the inclusion of autologous or heterologous hUCBP is an important and feasible alternative due to the increased number of public and private UCB banks of cryopreservation in the world. This aspect drove us to know the detailed composition of the hUCBP as well as of the CM where the hMSCs isolated from the UCT are expanded, by ^1^H-NMR and by Multiplexing LASER Bead Technology. ^1^H-NMR allowed for *in vitro* measurement of metabolites and the Multiplexing LASER Bead Technology using the bead analyzer Bio-Plex 200 allowed the quantification of numerous analyte categories such as cytokines, chemokines and growth factors.

Recent studies have shown that several obstetric and neonatal factors can affect the quality of UCB in terms of cell viability and CD34^+^ cell concentration [68] which is in accordance with the results obtained by flow cytometry concerning the CD34+ cell concentration and cell viability of the 11 samples of UCB used in this preliminary study. It is important to keep in mind the great variability of the UCB collected in terms of hematopoietic stem cells (CD34^+^ cells) and hMSCs concentration. This may have influenced the composition of the hUCBP considering the different components analyzed by ^1^H-NMR and the concentration of the growth factors, interleukins analyzed by Multiplexing LASER Bead Technology. The most important cause of the limited success of hMSC isolation from the UCB is the low frequency of these cells. Culture techniques have shown the presence of only 1–2 hMSCs clones per 10^8^ mononuclear cells of UCB, whereas in fresh bone marrow, hMSCs may account for 1–100 per million nucleated cells [69]. In addition, the newborn and the maternal factors are important for this individual variability, such as birth weight, which probably has a positive correlation with each of the clinical features of CD34^+^ cell number, TNC count and unit volume of UCB collected, followed by the placental weight. A longer gestational period would probably decrease CD34^+^ cell number. Female baby and mode of vaginal delivery of neonates were found to have larger amounts of TNC in UCB collected [68]. The signal for α-glucose and β-glucose are 5 or 6 fold higher in hUCBS analyzed when compared to CM. This is consistent with the function of the UCB to provide energetic support for the fetus during intrauterine development and due to the UCB collecting bag anticoagulant CPD content. One of the hUCBP (plasma sample #2) was contaminated with *Bacteroides distasonis* in anaerobiosis. This contamination did not interfere with the composition of the different components measured by ^1^H-NMR which was similar to the other samples analyzed by this method.

UCB and more recently UCT have been stored cryopreserved in private and public cord blood and tissue banks worldwide in order to obtain hematopoietic and hMSCs and, although guidelines exist (Netcord – Foundation for the Accreditation of Cellular Therapy), standardized procedures for UCB and UCT transport from the hospital/clinic to the laboratory, storage, processing, cryopreservation and thawing are not available. These may be critical in order to obtain a higher viable number of stem cells after thawing and to limit microbiological contamination. Some differences observed in the UCB samples analyzed by ^1^H-NMR might be due to the temperature oscillations during the transport from the hospital/clinic or due to different periods of time since the collection of the UCB and the arrival to the processing laboratory. Despite the evident individual variability of the UCB concerning the number of hematopoietic stem cells CD34^+^, the hUCBP has high concentrations of growth factors which are comparable to the CM where the hMSCs were cultured. Rodrigues et al. [57] proved that composite treatment with PDGF, FGF-2 and TGF-β1 appears to be a good alternative for proliferation in vitro to replace FBS. The massively high content of PDGFs and TGF-βs in hUCBP revealed in our cytokines array (Table S1 and Figure 2) may suggest that hUCBP supplemented with FGF-2 would be an ideal solution for FBS replacement. The high concentrations of RANTES (Table S3, 78903 pg/ml in hUCBS) that is a chemokine that promotes the recruitment and activation of inflammatory cells such as monocytes, lymphocytes, mast cells and eosinophils must also be highlighted from our data.

Considering the data obtained by ^1^H-NMR and Multiplexing LASER Bead Technology, which have proven to be adequate and complementary methods for an accurate composition analysis of the tested samples, the hUCBP might be an alternative for the FBS culture medium supplement used in hMSCs isolation, expansion and cryopreservation. This assumption is in line with other published research papers [70]–[73] where it was tested the culture medium replacement of FBS by hUCBP and the cell growth rate, specific biomarkers, and differentiation properties were evaluated to characterize the hMSCs proliferation and specific properties and it was concluded that hUCBP was probably superior to FBS in deriving and culturing the hMSCs from the umbilical cord Wharton's jelly. Also, the CM obtained by hMSCs expansion [5] and the hUCBP are very rich in growth factors with proliferative and anti-apoptotic functions, being an attractive alternative to allo-transplanting and xeno-transplanting of hMSCs for tissue regeneration. In the paper published by Lin et al (2013) [74] it was proved that CM from hMSCs could significantly improve the expansion of umbilical cord blood (UCB) CD34^+^ cells. It was demonstrated that post-thawing, the fold, percentage and colony forming unit numbers of CD34^+^ cells were significantly increased when it was used the CM for culture expansion while the percentages of apoptotic, necrotic, dead and sub-G1 phase cells were significantly decreased compared to controls. The results permitted to be hypothesized that these improvements were probably related to the high levels of cytokines, cell adhesion molecules and growth factors present in the CM that help to preserve cell membrane integrity during freezing and stimulate mitosis post-thaw. So, CM may be considered a useful supplement for freezing CD34^+^ cells in cord blood banks, like the hUCBP [74].

This research work also focus the possible use of hUCBP as a substitute for the FBS used in hMSCs cell culture and expansion. Further investigation involving a larger number of hUCBP samples must be performed using the ^1^H-NMR and Multiplexing LASER Bead analysis. The application of stem cell-derived biomolecules for tissue regeneration may provide alternative, stem cell-derived therapies that overcome current cell sourcing issues [2].

## Conclusions

The CM obtained by hMSCs expansion and the hUCBP are very rich in growth factors with proliferative and anti-apoptotic functions, being an attractive alternative to allo-transplanting and xeno-transplanting of hMSCs for tissue regeneration and to be used as culture medium supplement for hMSCs isolation, expansion and cryopreservation. The therapeutic use of CM and hUCBP seem to have a promising future in regenerative medicine by providing important growth factors. hUCBP should also be considered as a new strategy for supplementation of hMSCs cultures, mostly when the clinical application is intended.

## Supporting Information

Figure S1 **Schematic representation of the methods used for MSCs and hUCBS metabolic profile characterization.**
(TIF)

Table S1 **Quantitative distribution of the main metabolites (relative to internal TSP) ^1^H-NMR observed in the 600 MHz spectra of** ***DMEM*** **,** ***24 h DMEM*** **,** ***Com. Medium*** **,** ***24 h/48 h Com. Medium*** **and** ***Plasma*** **of the UCB (average values from samples 1**–**8).** *Not detectable (n.d.) due to peaks overlap and low intensity. - <10 pg/mL; ± ≧ 10 <100 pg/ml; + ≧ 100 <1000 pg/ml; ++ ≧ 1000 <2000 pg/ml; +++ ≧ 2000 <3000 pg/ml; ++++ ≧ 3000 <4000 pg/ml; +++++ ≧ 4000 <5000 pg/ml; ++++++ ≧ 5000 pg/ml; **O** Out of range.
(DOCX)

Table S2 **Relative quantitative distribution of the main metabolites observed in the 600 MHz ^1^H-NMR spectra of the different UCB plasma samples (1**–**8) analysed.** The ^1^H resonances used for the quantitative determination are pointed. * Not detectable (n.d.) due to peaks overlap and low intensity; ** Total integral of defined spectral area.
(DOCX)

Table S3 **Human Primary Cytokine Array/Chemokine Array 41-Plex Panel and TGF-β 3-Plex Array Multi-Species (Eve Technologies. Calgary, Alberta, Canada) for** ***UCB Plasma*** **, unconditioned (** ***Com. Medium*** **and** ***DMEM*** **) and conditioned media (** ***24/48 h Com. Medium*** **and** ***DMEM*** **).**
(DOCX)

Table S4 **^1^H-NMR chemical shifts and multiplicity of the resonance signals of the main metabolites (aminoacids and others) observed in the spectra of** ***DMEM*** **,** ***24 h DMEM*** **,** ***Com. Medium*** **, and** ***24 h/48 h Com. Medium*** **.**
(DOCX)

Table S5 **Relative ratio in terms of the integrals for signal intensity (obtained from ^1^H-NMR spectra) for** ***DMEM*** **,** ***24 h DMEM*** **,** ***Com. Medium*** **, and** ***24 h/48 h Com. Medium and*** **hUCBS samples (average).** Data normalized after citric acid values were removed. High values for citric acid were artificially induced upon hUCBS sample collection since it was used as an anticoagulant in the plasma containers.
(DOCX)

Table S6 **Normalized nomenclature for growth factor (GF) and cytokine including current abbreviations.**
(DOCX)
