# Supplementary figures and images for: MSCs Conditioned Media and Umbilical Cord Blood Plasma Metabolomics and Composition
Source: PLoS One. 2014 Nov 25;9(11):e113769. doi: 10.1371/journal.pone.0113769 (PMC4244191; doi:10.1371/journal.pone.0113769)

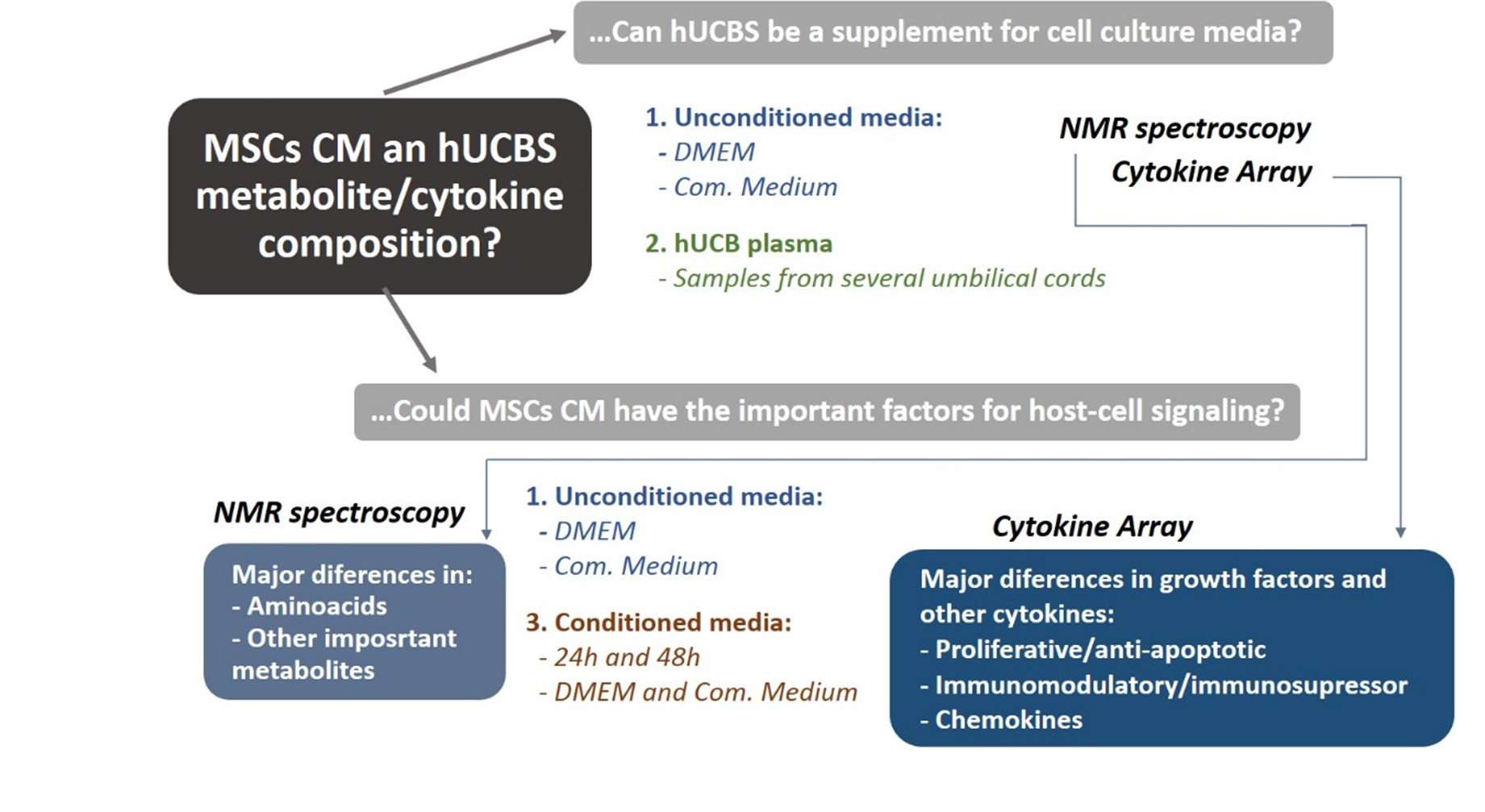

Supplement: Figure S1 — Schematic representation of the methods used for MSCs and hUCBS metabolic profile characterization. (TIF) [file pone.0113769.s001.tif]
