# Supplementary material for: MSCs Conditioned Media and Umbilical Cord Blood Plasma Metabolomics and Composition
Source: PLoS One. 2014 Nov 25;9(11):e113769. doi: 10.1371/journal.pone.0113769 (PMC4244191; doi:10.1371/journal.pone.0113769)
Supplement: Table S1 — Quantitative distribution of the main metabolites (relative to internal TSP) 1H-NMR observed in the 600 MHz spectra of DMEM , 24 h DMEM , Com. Medium , 24 h/48 h Com. Medium and Plasma of the UCB (average values from samples 1–8). *Not detectable (n.d.) due to peaks overlap and low intensity. - <10 pg/mL; ± ≧ 10 <100 pg/ml; + ≧ 100 <1000 pg/ml; ++ ≧ 1000 <2000 pg/ml; +++ ≧ 2000 <3000 pg/ml; ++++ ≧ 3000 <4000 pg/ml; +++++ ≧ 4000 <5000 pg/ml; ++++++ ≧ 5000 pg/ml; O Out of range. (DOCX) [file pone.0113769.s002.docx]

**Table S1**

|  |  | **DMEM** | **24h DMEM** | **48h DMEM** | **Com. Medium** | **24h Com. Medium** | **48h Com. Medium** | **UCB**  **Plasma** |
| --- | --- | --- | --- | --- | --- | --- | --- | --- |
| **Proloferatitive/**  **Anti-apoptosis** | **TGF-β1** | ± | - | ± | + | ++ | ++++ | ++++++ |
|  | **TGF-β2** | o | o | - | + | + | + | +++ |
|  | **TGF-β3** | o | o | o | o | o | - | + |
|  | **EGF** | o | - | - | o | - | - | ± |
|  | **FGF-2** | ± | ± | ± | ++++ | +++ | + | ± |
|  | **TGF-α** | - | - | - | - | - | - | ± |
|  | **G-CSF** | - | ± | ± | - | + | ++ | ± |
|  | **GM-CSF** | - | ± | ± | - | ± | ± | ± |
|  | **PDGF-AA** | - | - | - | - | - | - | ++ |
|  | **PDGF-BB** | o | o | - | o | o | o | +++++ |
|  | **VEGF** | ± | ± | ± | ± | ± | ± | ± |
|  |  |  |  |  |  |  |  |  |
|  |  | **DMEM** | **24h DMEM** | **48h DMEM** | **Com. Medium** | **24h Com. Medium** | **48h Com. Medium** | **UCB**  **Plasma** |
| **Chemokines** | **MCP-1** | - | ++++ | +++ | - | +++ | +++ | + |
|  | **MIP-1a** | o | - | - | o | - | - | - |
|  | **MIP-1b** | - | - | - | o | - | - | ± |
|  | **RANTES** | - | ± | ± | - | + | + | ++++++ |
|  | **MCP-3** | - | + | + | - | + | + | ± |
|  | **Eotaxin** | - | ± | ± | - | ± | ± | + |
|  | **Fractakline** | o | - | ± | ± | ± | ± | + |
|  | **GRO** | o | +++++ | ++++++ | o | ++++++ | ++++++ | + |
|  | **IL-6** | o | + | + | - | + | ++ | - |
|  | **IL-8** | - | +++++ | ++++++ | - | ++++++ | ++++++ | - |
|  | **IP-10** | o | ± | ± | o | ± | + | ± |
|  |  |  |  |  |  |  |  |  |
|  |  | **DMEM** | **24h DMEM** | **48h DMEM** | **Com. Medium** | **24h Com. Medium** | **48h Com. Medium** | **UCB**  **Plasma** |
| **Immunomodulatory/ Immunosupressors** | **TGF-β1** | ± | - | ± | + | ++ | ++++ | ++++++ |
|  | **TGF-β2** | o | o | - | + | + | + | +++ |
|  | **TGF-β3** | o | o | o | o | o | - | + |
|  | **IFN**γ | - | - | - | - | - | - | - |
|  | **IL-17A** | - | - | - | - | - | - | - |
|  | **IL-12p70** | o | o | - | o | - | - | - |
|  | **IL-10** | - | - | - | - | - | - | - |
|  | **IL-4** | o | - | o | o | - | - | o |
|  | **IL-13** | - | - | - | o | - | - | o |
