# Supplementary material for: MSCs Conditioned Media and Umbilical Cord Blood Plasma Metabolomics and Composition
Source: PLoS One. 2014 Nov 25;9(11):e113769. doi: 10.1371/journal.pone.0113769 (PMC4244191; doi:10.1371/journal.pone.0113769)
Supplement: Table S2 — Relative quantitative distribution of the main metabolites observed in the 600 MHz 1H-NMR spectra of the different UCB plasma samples (1–8) analysed. The 1H resonances used for the quantitative determination are pointed. * Not detectable (n.d.) due to peaks overlap and low intensity; ** Total integral of defined spectral area. (DOCX) [file pone.0113769.s003.docx]

**Table S2**

| **Metabolites** | **Group** | **Chemical shifts (ppm);** | **Plasma**  **Sample**  **#1** | **Plasma**  **Sample**  **#2** | **Plasma**  **Sample**  **#3** | **Plasma**  **Sample**  **#4** | **Plasma**  **Sample**  **#5** | **Plasma**  **Sample**  **#6** | **Plasma**  **Sample**  **#7** | **Plasma**  **Sample**  **#8** |
| --- | --- | --- | --- | --- | --- | --- | --- | --- | --- | --- |
| **Lipids** | CH_3_ | 0.87 | 3.01 | 3.71 | 3.69 | 0.67 | 1.78 | 1.96 | 1.97 | 2.83 |
| **Ile/Leu/Val** | CH_3_ | 0.9-1.0 | 0.61 | 0.67 | 0.61 | 0.21 | 0.42 | 0.41 | 0.45 | 0.55 |
| **β-HB** | CH_3_ | 1.19 | 2.90 | 1.59 | 3.73 | 2.29 | 0.38 | 1.15 | 1.03 | 2.41 |
| **Lipids** | CH_2_ | 1.28 | 5.17 | 5.04 | 5.36 | 1.23 | 2.30 | 2.37 | 2.38 | 5.11 |
| **Lactate** | CH_3_ | 1.34 | 8.87 | 9.10 | 10.03 | 3.48 | 7.21 | 9.24 | 4.63 | 6.73 |
| **Alanine/Lys** | CH_3_ | 1.48 | 1.20 | 1.15 | 1.16 | 0.36 | 1.40 | 1.26 | 1.01 | 0.42 |
| **Lys/Arg/Leu/Lip** | CH/CH_2_ | 1.6-1.8 | 1.33** | 1.84** | 1.76** | 1.03** | 1.35** | 1.80** | 1.32** | 1.32** |
| **Acetate** | CH_3_ | 1.92 | 0.23 | 0.37 | 2.50 | 0.37 | 0.19 | 0.24 | 0.22 | 0.34 |
| **Glu/Glu/Meth** | CH_2_ | 1.9-2.5 | n.d.* | n.d.* | n.d.* | n.d.* | n.d.* | n.d.* | n.d.* | n.d.* |
| **Piruvate** | CH_2_ | 2.39 | 0.32 | 0.31 | 0.30 | 0.13 | 0.13 | 0.16 | 0.22 | 0.32 |
| **Citric acid** | CH_2_ | 2.62 | 75.02 | 75.14 | 81.00 | 131.39 | 132.50 | 69.07 | 66.93 | 110.47 |
| **Inositol** | CH | 3.37 | 1.15 | 2.26 | 1.68 | 1.52 | 2.50 | 2.64 | 0.59 | 1.00 |
| **α-Glucose** | H1 | 5.24 | 25.85 | 26.68 | 36.35 | 43.36 | 40.02 | 25.88 | 25.12 | 42.83 |
| **β-Glucose** | H1 | 4.65 | 34.52 | 31.61 | 42.79 | 64.23 | 50.28 | 32.95 | 31.47 | 53.71 |
| **Tyrosine** | H3.5 | 6.90 | 0.11 | 0.12 | 0.10 | 0.06 | 0.09 | 0.11 | 0.10 | 0.19 |
| **Phenylalanine** | H4 | - | 0.10 | 0.13 | 0.15 | 0.08 | 0.09 | 0.19 | 0.18 | 0.20 |
| **Histidine** | H5 | 7.05 | 0.17 | 0.20 | 0.18 | 0.08 | 0.15 | 0.16 | 0.15 | 0.17 |
| **Nicotinamide** | H2 | 8.89 | n.d.* | n.d.* | n.d.* | n.d.* | n.d.* | n.d.* | n.d.* | n.d.* |
| **Tryptophan** | H4 | 7.74 | n.d.* | n.d.* | n.d.* | n.d.* | n.d.* | n.d.* | n.d.* | 0.06 |
| **Thiamine** | H12 | 8.03 | n.d.* | n.d.* | n.d.* | n.d.* | n.d.* | n.d.* | n.d.* | n.d.* |
| **Formate** | CH | 8.47 | 1.45 | 1.45 | 1.69 | 0.87 | 0.70 | 0.92 | 0.72 | 1.23 |
| **Urea** | NH_2_ | 5.90 | 0.19 | 0.13 | 0.11 | 0.11 | 0.14 | 0.10 | 0.12 | 0.10 |
| **Purines** | CH | 8.1-8.4 | 0.61** | 0.60** | 0.65** | 1.49** | 0.94** | 0.36** | 0.31** | 0.60** |
