# Supplementary material for: MSCs Conditioned Media and Umbilical Cord Blood Plasma Metabolomics and Composition
Source: PLoS One. 2014 Nov 25;9(11):e113769. doi: 10.1371/journal.pone.0113769 (PMC4244191; doi:10.1371/journal.pone.0113769)
Supplement: Table S3 — Human Primary Cytokine Array/Chemokine Array 41-Plex Panel and TGF-β 3-Plex Array Multi-Species (Eve Technologies. Calgary, Alberta, Canada) for UCB Plasma , unconditioned ( Com. Medium and DMEM ) and conditioned media ( 24/48 h Com. Medium and DMEM ). (DOCX) [file pone.0113769.s004.docx]

**Table S3**

|  | **UCB Plasma (M ± SD)** | **UCB Plasma #9** | **UBC Plasma #10** | **UCB Plasma #11** | **DMEM** | **24h DMEM** | **48h DMEM** | **Com. Medium** | **24h Com. Medium** | **48h Com. Medium** |
| --- | --- | --- | --- | --- | --- | --- | --- | --- | --- | --- |
|  | **pg/mL** | | | | | | | | | |
| **TGF-β1 *** | **16670.07 ± 6177.31** | 13582.18 | 23782.49 | 12645.54 | 11.7 | 2.44 | 46.15 | 523.76 | 1716.02 | 3162.8 |
| **TGF-β2 *** | **2832.07 ± 1095.11** | 2216.50 | 4096.45 | 2183.26 | NA | NA | 5.45 | 188.66 | 400.34 | 562.07 |
| **TGF-β3 *** | **182.51 ± 107.09** | 305.53 | 110.12 | 131.88 | NA | NA | NA | NA | NA | 7.75 |
| **EGF** | **67.81 ± 56.15** | 42.52 | 132.16 | 28.76 | NA | 2.32 | 3.43 | NA | 2.09 | 3.65 |
| **FGF-2** | **36.33 ± 21.22** | 35.38 | 58.00 | 15.60 | 13.48 | 62.93 | 68.69 | 3111.24 | 2373.14 | 637.44 |
| **Eotaxin** | **163.32 ± 36.73** | 186.06 | 182.96 | 120.95 | 1.85 | 17.13 | 62.6 | 1.24 | 27.41 | 46.38 |
| **TGF-α** | **10.74 ± 2.30** | 8.13 | 12.47 | 11.62 | 0.35 | 0.63 | 0.39 | 0.46 | 0.96 | 0.79 |
| **G-CSF** | **29.24 ± 21.31** | 42.65 | 4.66 | 40.40 | 0.64 | 42.11 | 91.76 | 2.32 | 302.48 | 1066.3 |
| **Flt-3L** | **NA** | NA | NA | NA | 1.60 | 3.83 | 4.24 | 2.65 | 9.07 | 5.86 |
| **GM-CSF** | **13.51 ± 1.28** | 14.93 | 12.45 | 13.16 | 0.02 | 10.28 | 18.85 | 1.21 | 44.78 | 92.73 |
| **Fractakline** | **110.62 ± 27.90** | 134.60 | 80.00 | 117.25 | NA | 7.9 | 32.58 | 45.15 | 60.69 | 32.58 |
| **IFNα2** | **35.41 ± 4.44** | 39.60 | 30.76 | 35.88 | 2.97 | 12.83 | 20.58 | 2.53 | 31.13 | 29.84 |
| **IFNγ** | **2.19 ± 0.20** | 2.41 | 2.15 | 2.02 | 0.43 | 3.34 | 4.6 | 0.71 | 3.55 | 4.11 |
| **GRO** | **586.58 ± 170.02** | 512.27 | 781.11 | 466.36 | NA | 4120.75 | 6192.28 | NA | 5867.99 | 6159.6 |
| **IL-10** | **^#^0.95 ± 1.29** | 1.86 | 0.03 | NA | 0.57 | 0.77 | 1.18 | 0.17 | 1.48 | 1.04 |
| **MCP-3** | **13.34 ± 5.69** | 15.27 | 6.94 | 17.81 | 6.39 | 141.32 | 529.11 | 1.6 | 253.29 | 683.52 |
| **IL-12 (p40)** | **^#^5.85 ± 4.48** | 9.02 | 2.68 | NA | 0.93 | 0.35 | 3.48 | 1.22 | 33.71 | 31.86 |
| **MDC** | **775.23 ± 333.28** | 587.04 | 1160.04 | 578.62 | 8.78 | 38.78 | 105.86 | 19.14 | 98.57 | 91.06 |
| **IL-12 (p70)** | **5.36 ± 1.73** | 6.05 | 3.39 | 6.63 | NA | NA | 6.45 | NA | 2.48 | 4.01 |
| **PDGF-AA** | **1155.70 ± 541.92** | 969.91 | 1766.08 | 731.12 | 0.05 | 0.34 | 0.59 | 0.06 | 0.75 | 0.61 |
| **IL-13** | **NA** | NA | NA | NA | 0.13 | 0.59 | 0.63 | NA | 1.04 | 0.47 |
| **PDGF-BB** | **4958.14 ± 1553.58** | 3724.41 | 6702.86 | 4447.15 | NA | NA | 0.47 | NA | NA | NA |
| **IL-15** | **^#^0.73 ± 0.38** | NA | 1.00 | 0.46 | NA | 0.64 | 2.00 | 0.52 | 1.75 | 2.75 |
| **sCD40L** | **5950.46 ± 6180.38** | 5081.56 | 12519.31 | 250.51 | 2.54 | 2.76 | 6.49 | 1.88 | 7.15 | 14.15 |
| **IL-17A** | **1.81 ± 0.17** | 1.66 | 1.77 | 2.00 | 0.54 | 0.98 | 1.8 | 0.64 | 2.19 | 2.39 |
| **IL-1RA** | **62.28 ± 46.92** | 69.80 | 104.99 | 12.05 | 4.06 | 29.21 | 53.53 | 1.14 | 47.03 | 62.36 |
| **IL-1α** | **4.75 ± 2.57** | 6.23 | 1.78 | 6.23 | NA | 25.65 | 45.02 | 0.58 | 11.87 | 22.78 |
| **IL-9** | **0.31 ± 0.06** | 0.24 | 0.36 | 0.32 | 0.26 | 0.57 | 0.55 | 0.24 | 0.94 | 1.03 |
| **IL-1β** | **^##^1.15** | NA | 1.15 | NA | NA | 1.87 | 2.30 | 0.44 | 1.65 | 3.18 |
| **IL-2** | **^##^0.06** | NA | 0.06 | NA | NA | 1.80 | 2.18 | NA | 1.03 | 1.99 |
| **IL-3** | **^#^0.32 ± 0.37** | 0.58 | NA | 0.05 | NA | NA | 1.01 | NA | 0.78 | 1.41 |
| **IL-4** | **NA** | NA | NA | NA | NA | 2.43 | NA | NA | 1.73 | 4.4 |
| **IL-5** | **0.27 ± 0.09** | 0.24 | 0.20 | 0.37 | 0.15 | 0.15 | 0.22 | 0.12 | 0.44 | 0.21 |
| **IL-6** | **0.29 ± 0.25** | 0.06 | 0.26 | 0.55 | NA | 262.74 | 607.09 | 0.29 | 909.52 | 1368.87 |
| **IL-7** | **0.66 ± 0.15** | 0.83 | 0.60 | 0.56 | 0.17 | 1.35 | 1.39 | 0.07 | 1.33 | 2.02 |
| **IL-8** | **9.94 ± 0.95** | 9.10 | 9.76 | 10.97 | 0.46 | 4240.55 | 8075.26 | 2.7 | 7312.85 | 8151.12 |
| **IP-10** | **75.55 ± 15.09** | 77.78 | 89.41 | 59.47 | NA | 34.76 | 48.69 | NA | 67.99 | 172.27 |
| **MCP-1** | **523.92 ± 206.94** | 620.52 | 286.35 | 664.90 | 5.36 | 3784.04 | 2704.17 | 4.82 | 2414.15 | 2612.39 |
| **MIP-1α** | **6.63 ± 2.15** | 8.50 | 7.12 | 4.28 | NA | 1.95 | 3.36 | NA | 0.58 | 8.71 |
| **MIP-1β** | **31.44 ± 14.60** | 47.37 | 18.70 | 28.25 | 0.35 | 2.03 | 6.13 | NA | 4.2 | 4.2 |
| **RANTES**** | **78903.24 ± 38534.68** | 82762.07 | 115363.32 | 38584.32 | 3.92 | 54.54 | 89.99 | 2.83 | 140.32 | 252.06 |
| **TNFα** | **25.25 ± 2.90** | 23.07 | 24.14 | 28.54 | 0.01 | 0.22 | 0.64 | 0.27 | 0.45 | 0.55 |
| **TNFβ** | **^##^0.67** | 0.67 | NA | NA | NA | 0.22 | 0.64 | 0.27 | 0.45 | 0.55 |
| **VEGF** | **99.06 ± 52.75** | 129.14 | 129.89 | 38.15 | 12.25 | 19.06 | 18.58 | 11.36 | 16.85 | 22.06 |

* Dilution Factor = 30 (values corrected to dilution applied to sample upon testing)

** Dilution Factor = 100 (values corrected to dilution applied to sample upon testing)

NA: not applicable, values below detection range

#: two samples below detection range, value corresponding to one sample only

##: one sample below detection range, value corresponding to two samples only
