# Supplementary material for: MSCs Conditioned Media and Umbilical Cord Blood Plasma Metabolomics and Composition
Source: PLoS One. 2014 Nov 25;9(11):e113769. doi: 10.1371/journal.pone.0113769 (PMC4244191; doi:10.1371/journal.pone.0113769)
Supplement: Table S4 — 1H-NMR chemical shifts and multiplicity of the resonance signals of the main metabolites (aminoacids and others) observed in the spectra of DMEM , 24 h DMEM , Com. Medium , and 24 h/48 h Com. Medium . (DOCX) [file pone.0113769.s005.docx]

**Table S4:** ^1^H-NMR chemical shifts and multiplicity of the resonance signals of the main metabolites (aminoacids and others) observed in the spectra of *DMEM*, *24h DMEM*, *Com. Medium*, and *24h/48h* *Com. Medium*.

| **Metabolites** | **Group** | **Chemical shifts (ppm);**  **Multiplicity** | **DMEM** | **24h DMEM** | **Com. Medium** | **24h Com. Medium** | **48h Com. Medium** |
| --- | --- | --- | --- | --- | --- | --- | --- |
| **Amino Acids** | | | | | | | |
| **Alanine** | βCH_3_  αCH | 1.48(d)  3.78(q) | - | - | 4.639 | 2.57 | 3.535 |
| **Arginine** | δCH_2_  γCH_2_  βCH_2_  αCH | 3.23(t)  1.67(m)  1.89(m)  3.77(t) | 0.309 | 0.389 | 0.523 | 0.46 | 0.514 |
| **Cysteine** | βCH_2_  αCH | 3.17/2.93**  3.80 | n.d.* | n.d.* | n.d.* | n.d.* | n.d.* |
| **Cystine** | βCH_2_  αCH | 3.17/2.93**  3.77 | n.d.* | n.d.* | n.d.* | n.d.* | n.d.* |
| **Glutamate** | γCH_2_  βCH_2_  αCH | 2.36(t)  2.08(m)  4.18(dd) | - | - | 2.201 | 1.73 | 2.106 |
| **Glutamine** | γCH_2_  βCH_2_  αCH | 2.44(t)  2.51/2.04(m)  4.18(dd) | 1.770 | 2.410 | 3.233 | 2.48 | 2.048 |
| **Isoleucine** | δCH_3_  γCH_2_  βCH  βCH_3_  αCH | 0.95(dd)  1.27/1.46(m)  1.98(m)  1.01(d)  3.67(d) | 0.560 | 0.670 | 1.443 | 1.18 | 1.367 |
| **Leucine** | δCH_3_  δ’CH_3_  γCH  βCH_2_  αCH | 0.95(d)  0.97(d)  1.69(m)  1.71(m)  3.73(d) | 0.490 | 0.545 | 1.439 | 0.95 | 1.361 |
| **Lysine** | εCH_2_  δCH_2_  γCH_2_  βCH_2_  αCH | 3.04(t)  1.73(m)  1.47(m)  1.93(m)  3.77(t) | 0.610 | 0.718 | 2.019 | 1.21 | 1.618 |
| **Methionine** | δCH_3_  γCH_2_  βCH_2_  αCH | 2.2 (s)  2.45(m)  2.13(m)  3.75(t) | 0.380 | 0.390 | 7.063 | 3.00 | 3.031 |
| **Thiamine** | H6  H12 | 5.47  8.03 | 0.009 | 0.0098 | 0.0089 | 0.01 | 0.009 |
| **Threonine** | γCH_3_  βCH_2_  αCH | 1.33(d)  4.25(m)  3.56(d) | 0.470 | 0.654 | 1.651 | 1.34 | 1.523 |
| **Tryptophan** | βCH_2_  αCH  H2  H4  H5  H6  H7 | 3.29/3.47**  4.05  7.30  7.74(d)  7.20(t)  7.28(t)  7.55(d) | 0.060 | 0.060 | 0.068 | 0.06 | 0.059 |
| **Valine** | γCH_3_  γ’CH_3_  βCH_2_  αCH | 0.98(d)  1.05(d)  2.27(m)  3.61(d) | 0.530 | 0.634 | 1.421 | 0.94 | 1.338 |

**Table S4** (continuation).

| **Metabolites** | **Group** | **Chemical shifts (ppm);**  **Multiplicity** | **DMEM** | **24h DMEM** | **Com. Medium** | **24h Com. Medium** | **48h Com. Medium** |
| --- | --- | --- | --- | --- | --- | --- | --- |
| **Others** |  |  |  |  |  |  |  |
| **α-Glucose** | H1  H2  H3  H4  H5  H6a  H6b | 5.24(d)  3.54(dd)  3.72(t)  3.41(t)  3.47(ddd)  3.91(dd)  3.72(dd) | 7.110 | 8.194 | 5.077 | 2.270 | 3.727 |
| **β-Glucose** | H1  H2  H3  H4  H5,6a  H6b | 4.65(d)  3.24(t)  3.49(t)  3.42(t)  3.84(m)  3.78(dd) | 7.100 | 10.069 | 6.753 | 2.683 | 4.515 |
| **Acetate** | CH_3_COO^-^ | 1.92(s) | 0.136 | 0.136 | 0.468 | 0,300 | 0.237 |
| **Ethanol** | βCH_3_  αCH_2_ | 1.18(t)  3.65(q) | - | 0.419 | 0.146 | 0.511 | 0.470 |
| **Formate** | CH | 8.47 | 0.010 | 0.030 | 0.060 | 0.050 | 0.06 |
| **Lactate** | βCH_3_  αCH | 1.34(d)  4.13(q) | - | 2.636 | 3.045 | 6.238 | 5.085 |
| **Nicotinamide** | H2  H4  H5  H6 | 8.89(s)  8.21(d)  7.57(t)  8.69(d) | 0.030 | 0.027 | 0.056 | 0.043 | 0.050 |
| **Purines** | CH | 8.1-8.4 |  |  |  |  |  |
| **Piruvate** | CH_2_ | 2.38(s) | - | - | 1.186 | 0.457 | 0.673 |
| **Urea** | NH_2_ | 5.90 | - | - | - | 0.040 | 0.090 |

* Not detectible (n.d.) due to peaks overlap and low intensity;

** Chem. Shifts from the literature.
