# Supplementary material for: MSCs Conditioned Media and Umbilical Cord Blood Plasma Metabolomics and Composition
Source: PLoS One. 2014 Nov 25;9(11):e113769. doi: 10.1371/journal.pone.0113769 (PMC4244191; doi:10.1371/journal.pone.0113769)
Supplement: Table S5 — Relative ratio in terms of the integrals for signal intensity (obtained from 1H-NMR spectra) for DMEM , 24 h DMEM , Com. Medium , and 24 h/48 h Com. Medium and hUCBS samples (average). Data normalized after citric acid values were removed. High values for citric acid were artificially induced upon hUCBS sample collection since it was used as an anticoagulant in the plasma containers. (DOCX) [file pone.0113769.s006.docx]

**Table S5**

| **Code** | **Metabolites** | **Group** | **Chemical shifts (ppm)** | **DMEM** | **24h DMEM** | **Com. Medium** | **24h Com. Medium** | **48h Com. Medium** | **Plasma**  **(average)** |
| --- | --- | --- | --- | --- | --- | --- | --- | --- | --- |
| **1** | Lipids | CH_3_ | 0.87 | - | - | - | - | - | 2.46 |
| **2** | Ile/Leu/Val | CH_3_ | 0.9-1.0 | 1.58 | 1.85 | 4.30 | 2.96 | 4.07 | 0.49 |
| **3** | Ethanol | CH_3_ | 1.18 | - | 0.42 | 0.15 | 0.51 | 0.47 | - |
| **4** | β-HB | CH_3_ | 1.19 | - | - | - | - | - | 1.93 |
| **5** | Lipids | CH_2_ | 1.28 | - | - | - | - | - | 3.62 |
| **6** | Threonine | CH_3_ | 1.33 | 0.47 | 0.65 | 1.65 | 1.275 | 1.52 | - |
| **7** | Lactate | CH_3_ | 1.34 | - | 2.64 | 3.05 | 6.24 | 5.09 | 7.41 |
| **8** | Alanine | CH_3_ | 1.48 | - | - | 4.64 | 2.475 | 3.54 | 1.00 |
| **9** | Lys/Arg | CH/CH_2_ | 1.6-1.8 | 0.92 | 1.11 | 2.52 | 1.645 | 2.13 | 1.47 |
| **10** | Acetate | CH_3_ | 1.92 | 0.14 | 0.14 | 0.47 | 0.3 | 0.24 | 0.56 |
| **11** | Glutamate | CH_2_ | 2.36 | - | - | 2.20 | 1.295 | 2.11 | n.d.* |
| **12** | Glutamine | CH_2_ | 2.51 | 1.77 | 2.41 | 3.23 | 2.39 | 2.05 | n.d.* |
| **13** | Methionine | CH_2_ | 2.45 | 0.38 | 0.39 | 7.06 | 2.88 | 3.03 | n.d.* |
| **14** | Piruvate | CH_2_ | 2.39 | - | - | 1.19 | 0.455 | 0.67 | 0.24 |
| **15** | Citric acid | CH_2_ | 2.62 | - | - | - | - | - | 92.69 |
| **16** | Choline | NCH_3_ | 3.21 | - | 0.01 | 0.09 | 0.065 | 0.09 | n.d.* |
| **17** | Inositol | CH | 3.37 | - | - | - | - | - | 1.67 |
| **18** | α-Glucose | H1 | 5.24 | 7.11 | 8.19 | 5.08 | 2.27 | 3.73 | 33.26 |
| **19** | β-Glucose | H1 | 4.65 | 7.10 | 10.07 | 6.75 | 2.685 | 4.52 | 42.70 |
| **20** | Tyrosine | H3.5 | 6.90 | 0.31 | 0.37 | 0.79 | 0.455 | 0.63 | 0.11 |
| **21** | Phenylalanine | H4 | - | 0.30 | 0.38 | 0.85 | 0.455 | 0.66 | 0.14 |
| **22** | Histidine | H5 | 7.05 | 0.16 | 0.17 | 0.42 | 0.235 | 0.30 | 0.16 |
| **23** | Nicotinamide | H2 | 8.89 | 0.03 | 0.03 | 0.06 | 0.04 | 0.05 | n.d.* |
| **24** | Tryptophan | H4 | 7.74 | 0.06 | 0.06 | 0.07 | 0.055 | 0.06 | 0.06 |
| **25** | Thiamine | H12 | 8.03 | 0.01 | 0.01 | 0.01 | 0.01 | 0.01 | n.d.* |
| **26** | Formate | CH | 8.47 | 0.01 | 0.03 | 0.06 | 0.05 | 0.06 | 1.13 |
| **27** | Urea | NH_2_ | 5.90 | - | - | - | 0.04 | 0.09 | 0.12 |
| **28** | Purines | CH | 8.1-8.4 | - | - | - | - | - | 0.70 |
