# Supplementary material for: MSCs Conditioned Media and Umbilical Cord Blood Plasma Metabolomics and Composition
Source: PLoS One. 2014 Nov 25;9(11):e113769. doi: 10.1371/journal.pone.0113769 (PMC4244191; doi:10.1371/journal.pone.0113769)
Supplement: Table S6 — Normalized nomenclature for growth factor (GF) and cytokine including current abbreviations. (DOCX) [file pone.0113769.s007.docx]

**Table S6**

| GF / Chemokine AbBreviation | Aliases | Growth Factor / Chemokine Designation |
| --- | --- | --- |
| TGF-β1 | **LAP** | Tranforming growth factor –beta 1/ Latency Associated Peptide |
| TGF-β2 | **G-TSF** | Tranforming growth factor –beta 2/ Glioblastoma-derived T-cell suppressor factor |
| TGF-β3 |  | Tranforming growth factor –beta 3 |
| EGF |  | Epidermal growth factor |
| FGF-2 | **bFGF/** **FGF-β** | Fibroblast growth factor – 2 / basic fibroblast growth factor/ fibroblast growth factor – beta |
| Eotaxin | **CCL11** | Eotaxin/ chemokine (c-c motif) ligand 11 |
| TGF-α |  | Tranforming growth factor - alfa |
| G-CSF | **CSF-3** | Granulocyte colony-stimulating factor /colony stimulating factor -3 |
| Flt-3L |  | Fms-like tyrosine kinase-3 ligand |
| GM-CSF | **CSF-2** | Granulocyte-macrophage colony-stimulating factor /colony stimulating factor -2 |
| Fractakline | **CX3CL1** | Fractakline/ chemokine (C-X3-C motif) ligand 1 |
| IFNα2 |  | Interferon alfa 2 |
| IFNγ | **IFG** | Interferon gamma/ Interferon gamma |
| GRO | **CXCL1-3/** **MGSA** | Growth-related oncogene/ chemokine (C-X-C motif) ligand 1-3/ melanoma growth-stimulating activity |
| IL-10 | **CSIF** | Interleukin-10 /human cytokine synthesis inhibitory factor |
| MCP-3 | **CCL7** | Monocyte chemoattractant protein -3/ chemokine (c-c motif) ligand 7 |
| IL-12 (p40) | **IL-12B/NKSF-2/CLMF-2** | Interleukin - 12 (p40) or B/ natural killer cell stimulatory factor 2/ cytotoxic lymphocyte maturation factor 2 |
| MDC | **CCL22** | Macrophage derived chemokine/ chemokine (c-c motif) ligand 22 |
| IL-12 (p70) | **IL-12A/NKSF-1/CLMF-1** | interleukin – 12(p70) or A/ natural killer cell stimulatory factor 1/ cytotoxic lymphocyte maturation factor 1 |
| PDGF-AA |  | Platelet derived growth factor -AA |
| IL-13 | **P600** | Interleukin – 13 |
| PDGF-BB |  | Platelet derived growth factor -BB |
| IL-15 |  | Interleukin – 15 |
| sCD40L |  | Platelet-derived soluble CD40 ligand |
| IL-17A | **CTLA8** | Interleukin – 17/ Cytotoxic T-lymphocyte-associated antigen 8 |
| IL-1RA | **IRAP** | Interleukin-1 receptor antagonista/ Interleukin-1 Receptor Antagonist Protein |
| IL-1α |  | Interleukin -1 alpha |
| IL-9 |  | Interleukin - 9 |
| IL-1β |  | Interleukin -1 beta |
| IL-2 | **TCGF** | Interleukin – 2/ T cell growth factor |
| IL-3 | **MCGF/MULTI-CSF** | Interleukin – 3/ mast cell growth factor/multi- colony stimulating factor |
| IL-4 | **BCGF -1/ BSF-1** | Interleukin – 4/ B cell growth factor -1/ B-cell stimulating factor-1 |
| IL-5 | **EDF** | Interleukin – 5/ eosinophil differentiation factor |
| IL-6 | **IFNβ2/BSF-2/HGF** | Interleukin – 6/ Interferon beta 2/ B-cell stimulating factor-2/ hepatocyte growth factor |
| IL-7 | **PBGF** | Interleukin – 7/ Pre-B-cell growth factor |
| IL-8 | **CXCL8/ MONAP** | Interleukin 8/ chemokine (C-X-C motif) ligand 8/ Monocyte-derived neutrophil-activating peptide |
| IP-10 | **CXCL10/ SCYB10** | Interferon gamma-induced protein 10/ chemokine (C-X-C motif) ligand 10/ small-inducible cytokine B10 |
| MCP-1 | **CCL2/ SCYA2** | monocyte chemotactic protein 1/ chemokine (C-C motif) ligand 2/ small inducible cytokine A2 |
| MIP-1α | **CCL3/ SCYA3** | Macrophage inflammatory proteins 1 alpha/ chemokine (C-C motif) ligand 3/ small inducible cytokine A3 |
| MIP-1β | **CCL4/ SCYA4** | Macrophage inflammatory proteins 1 beta/ chemokine (C-C motif) ligand 4/ small inducible cytokine A4 |
| RANTES | **CCL5/SCYA5** | Regulated on activation, normal T cell expressed and secreted/ chemokine (C-C motif) ligand 5/ small inducible cytokine A5 |
| TNFα | **TNF** | Tumor necrosis factor alfa/ tumor necrosis factor |
| TNFβ | **LTα** | Tumor necrosis factor beta/ lymphotoxin-alpha |
| VEGF | **VPF** | Vascular endothelial growth factor/ vascular permeability factor |
